# Soil temperature effects on the structure and diversity of plant and invertebrate communities in a natural warming experiment

**DOI:** 10.1111/1365-2656.12798

**Published:** 2018-02-13

**Authors:** Sinikka I. Robinson, Órla B. McLaughlin, Bryndís Marteinsdóttir, Eoin J. O'Gorman

**Affiliations:** ^1^ Department of Life Sciences Imperial College London Ascot UK; ^2^ Faculty of Biological and Environmental Sciences University of Helsinki Lahti Finland; ^3^ Agroécologie AgroSup Dijon INRA Université Bourgogne Franche‐Comté Dijon France; ^4^ Institute of Life and Environmental Sciences University of Iceland Reykjavík Iceland; ^5^ The Soil Conservation Service of Iceland Hella Iceland

**Keywords:** Arctic, climate change, Hengill, invertebrate community, natural experiment, pitfall, soil temperature, vegetation

## Abstract

1. Global warming is predicted to significantly alter species physiology, biotic interactions and thus ecosystem functioning, as a consequence of coexisting species exhibiting a wide range of thermal sensitivities. There is, however, a dearth of research examining warming impacts on natural communities.
2. Here, we used a natural warming experiment in Iceland to investigate the changes in above‐ground terrestrial plant and invertebrate communities along a soil temperature gradient (10°C–30°C).
3. The α‐diversity of plants and invertebrates decreased with increasing soil temperature, driven by decreasing plant species richness and increasing dominance of certain invertebrate species in warmer habitats. There was also greater species turnover in both plant and invertebrate communities with increasing pairwise temperature difference between sites. There was no effect of temperature on percentage cover of vegetation at the community level, driven by contrasting effects at the population level.
4. There was a reduction in the mean body mass and an increase in the total abundance of the invertebrate community, resulting in no overall change in community biomass. There were contrasting effects of temperature on the population abundance of various invertebrate species, which could be explained by differential thermal tolerances and metabolic requirements, or may have been mediated by changes in plant community composition.
5. Our study provides an important baseline from which the effect of changing environmental conditions on terrestrial communities can be tracked. It also contributes to our understanding of why community‐level studies of warming impacts are imperative if we are to disentangle the contrasting thermal responses of individual populations.

## Graphical abstract

The authors use a broad temperature gradient provided by indirect geothermal heating of the soil to show that diversity of plant and invertebrate communities decreases with warming. Moreover, invertebrate mean body size decreases and abundance increases, mediated by changes in plant community composition and differences in thermal tolerances of individual populations.

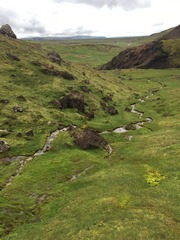

## 1 INTRODUCTION

The average global surface temperature has increased by 0.8°C since 1880 and is predicted to rise by at least 1.5°C during the next century (IPCC, 2014). Evidence for the ecological impacts of global warming at the population level is already substantial (see Parmesan & Yohe, 2003; Walther, 2010 for review), for example altered geographical distributions (Chen, Hill, Ohlemuller, Roy, & Thomas, 2011), shifts in phenology (Menzel, Sparks, & Estrella, 2006) and decreasing body size (Daufresne, Lengfellner, & Sommer, 2009). Species do not exist in isolation, however, and complex networks of trophic interactions make it difficult to extrapolate these population‐level impacts to the community or ecosystem levels (Ings et al., 2009; Walther, 2010; Woodward, Perkins, & Brown, 2010).

Understanding and quantifying the impacts of warming across multiple levels of biological organisation is important for modelling the ecological and evolutionary dynamics of ecosystem change (Montoya & Raffaelli, 2010). Climate change is already generating new communities and restructuring assemblages of species as a consequence of shifts in geographical distributions and/or adaptation to novel climatic conditions (Lurgi, López, & Montoya, 2012). It is likely that coexisting species will have a wide range of thermal sensitivities, as well as physiological and behavioural adaptations, inducing differential responses to warming and precipitating effects on the physiology of individuals, biotic interactions and ecosystem functioning. Warming should thus have effects across all levels of biological organisation, rooted in the relationship between temperature, metabolism and body mass (Brose et al., 2012; Brown, Gillooly, Allen, Savage, & West, 2004).

Cold‐adapted populations are likely to decline dramatically in abundance or become locally extinct with warming (Hering et al., 2009; Somero, 2010; Thomas et al., 2004). This is expected to result in decreasing α‐diversity, either through a decline in species richness and/or a decline in evenness, as more resilient populations with higher thermal optima begin to dominate the community (O'Gorman et al., 2012; Sharp et al., 2014). Such changes may be complicated if new, warm‐adapted species invade to offset the loss of cold‐adapted species (Krajick, 2004; Lejeusne, Chevaldonne, Pergent‐Martini, Bourdouresque, & Perez, 2010; Walther et al., 2002). This could maintain overall species richness, but the implications for evenness of the community are more difficult to predict (Andrew & Hughes, 2005; Friberg et al., 2009; Hillebrand, Soininen, & Snoeijs, 2010; Woodward, Dybkjaer, et al., 2010). For example, the decline of rove beetles with warming of an agroecosystem was balanced by increasing dominance of ground beetles, resulting in no overall effect of temperature on species richness (Berthe, Derocles, Lunt, Kimball, & Evans, 2015).

Consumer responses to temperature will be driven by resource availability. There is evidence for increasing primary production as a consequence of warming which may be particularly apparent in colder, high latitude areas, where earlier snowmelt and warmer conditions stimulate growth (Klanderud & Totland, 2005; Rustad et al., 2001). This increased productivity may offset the predicted reduction in plant biomass with warming due to stronger grazing pressure, thus supporting the higher metabolic demands of grazing species in a warmer environment. For example, warming and thus earlier snowmelt increased insect herbivore species richness and damage to several plant species in a long‐term manipulation experiment (Roy, Güsewell, & Harte, 2004). Plant diversity and community composition are likely to change as a consequence of warming, however (Klein, Harte, & Zhao, 2004; Walker et al., 2006), with evidence for a shift in plant dominance hierarchies and species evenness as a consequence of different thermal tolerances (Klanderud & Totland, 2005). Such changes in vegetation could alter habitat complexity and thus the associated invertebrate community, with plant and insect diversity shown to be positively correlated (Muren, Hoffmann, & Kwak, 2003).

Warming impacts on community composition will be mediated by the physiology of populations. In particular, body size underlies community‐ and population‐level responses to warming because it is directly shaped by temperature‐dependent metabolic processes (Brose et al., 2012; Brown et al., 2004). Communities are generally predicted to have a smaller mean body size in warmer regions either because they are made up of smaller sized species, or because the body size of species’ populations within the community are smaller (Daufresne et al., 2009). There is widespread empirical support for declining body mass with warming at the community and population levels (see Daufresne et al., 2009; Kingsolver & Huey, 2008 for reviews). The mechanisms underlying these patterns remain contentious (see Gardner, Peters, Kearney, Joseph, & Heinsohn, 2011; Klok & Harrison, 2013), with thermoregulation in endotherms (Porter & Kearney, 2009), “rate‐size” trade‐offs in ectotherms (DeLong, 2012) and competition for limiting nutrients in unicellular algae (Reuman, Holt, & Yvon‐Durocher, 2014) among the explanations. These models are not universally accepted and exceptions exist, for example in Baltic phytoplankton (Rüger & Sommer, 2012), centipedes (Vedel, Chipman, Akam, & Arthur, 2008), grasshoppers (Walters & Hassall, 2006), stream invertebrates (O'Gorman et al., 2012) and freshwater diatoms (Adams et al., 2013). Nevertheless, warming is predicted to alter the size‐structure of communities, with far‐reaching impacts on biotic interactions and ecosystem functioning (Montoya & Raffaelli, 2010).

Warming is predicted to reduce the standing stock of primary producers and herbivores due to the greater metabolic demands of consumer species in warmer environments (O'Connor, Gilbert, & Brown, 2011), although this may be offset by greater primary production. Consumer losses may be further compounded by ingestion inefficiency due to the weaker temperature dependence of feeding relative to metabolism (Lemoine & Burkepile, 2012; Vucic‐Pestic, Ehnes, Rall, & Brose, 2011). Larger organisms at the highest trophic levels are likely to be the most susceptible as a consequence of their greater metabolic demands and lower population densities (Brown et al., 2004; Petchey, McPhearson, Casey, & Morin, 1999). Loss of the largest species and the increased prevalence of smaller organisms at lower trophic levels should lead to an overall decline in community biomass (Yvon‐Durocher et al., 2011). The heterogeneity of species thermal responses or physiological characteristics that determine susceptibility to warming could, however, lead to variable effects on individual populations (Dollery, Hodkinson, & Jónsdóttir, 2006; Dong, Hou, Ouyang, & Zhang, 2013; O'Gorman et al., 2012).

Here, we make use of a high latitude natural soil warming experiment to investigate the impact of environmental temperature on the diversity, size‐structure, and biomass of terrestrial plant and invertebrate communities. We tested the following four hypotheses: (1) α‐diversity of plants and invertebrates decreases with increasing soil temperature; (2) species turnover of plants and invertebrates increases with increasing pairwise temperature difference between sites; (3) the mean body mass of invertebrates declines with increasing soil temperature at the community‐ and population levels**;** and (4) percentage cover of vegetation and the total abundance and biomass of invertebrates decrease with increasing soil temperature at the community level, with variable effects at the population level.

## 2 MATERIALS AND METHODS

### 2.1 Study site description

Geothermal regions that are not confounded by extreme physical or chemical variables have been identified as ideal systems for studying the impacts of environmental temperature on naturally occurring communities (O'Gorman et al., 2014). The Hengill valley in Iceland (64.03°N 21.18°W) contains a catchment of 16 geothermally heated streams that have been extensively studied and monitored over the past decade. Freshwater research at Hengill revealed a decline in macroinvertebrate evenness with increasing stream temperature and increased species turnover along the stream temperature gradient, but no effect of temperature on species richness (Friberg et al., 2009; Woodward, Perkins, et al., 2010). Studies have also shown mixed effects of stream temperature on population‐level body mass (Adams et al., 2013; O'Gorman et al., 2012) and increased community‐level abundance and biomass with increasing temperature (Hannesdottir, Gislason, Olafsson, Olafsson, & O'Gorman, 2013; O'Gorman et al., 2012, 2016).

**Figure 1:**
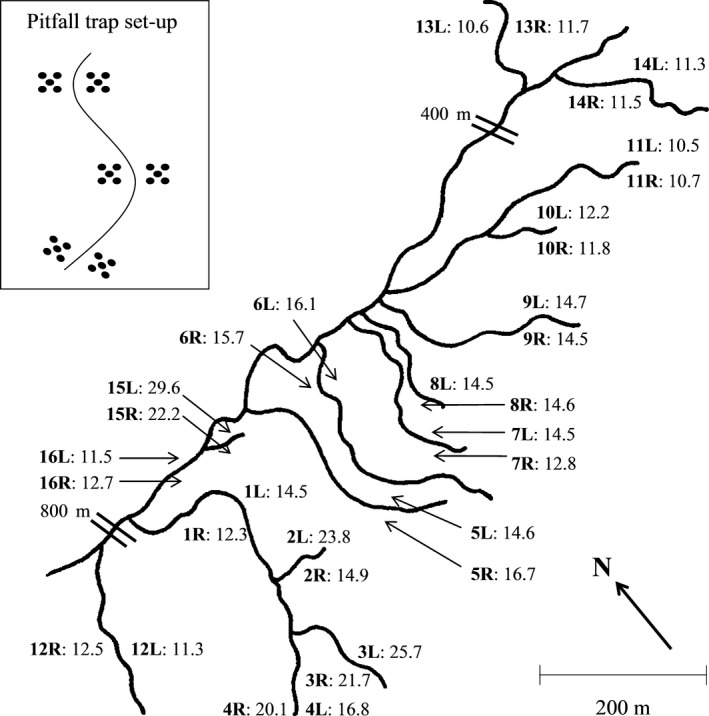
The 32 terrestrial sites in the Hengill geothermal region. Site names are annotated on the map in bold, consisting of a number (corresponding to the stream names in previous publications from the system) and a letter (corresponding to the bank of the stream: L = left; R = right). Each site name is followed by the mean soil temperature (°C) as calculated from 48‐hr temperature logger data in July 2013. The “=” symbol, used at two points along the main river, indicates a much greater distance than shown on the map. Three habitat patches were selected along the stream bank at each terrestrial site. Note that habitat patches were established directly opposite each other on the left and right bank of each stream (see inset). Five pitfall traps were established at each habitat patch in a 1 m^2^ area, located *c*. 30 cm from the stream bank, as shown in the inset

The various stream temperatures arise from differential geothermal heating of the landscape, which also creates a soil temperature gradient. Here, we chose three locations on each bank of the 16 streams previously studied at Hengill for a total of 96 habitat patches. Note that we consider the three locations on each bank as pseudoreplicates and so we present mean values from these three habitat patches for all environmental and biotic data for a total of 32 independent sites (Figure 1). GPS coordinates and spot measurements of soil temperature at 10 cm depth were taken between 12.00 hr and 18.00 hr on the same day at each habitat patch during pilot work in August 2012 using a digital soil thermometer (Digitron FM15). Soil temperature was monitored at each habitat patch during the main study period in July 2013 using Maxim Integrated DS1921G Thermocron iButton temperature loggers. One iButton was buried 10 cm below the soil surface at each site and the mean (±1 *SD*) temperature for each bank was calculated from measurements recorded every 10 min over a 48‐hr time period, that is including both night and day (Table S1).

### 2.2 Soil analyses

Soil moisture was measured at each habitat patch using a soil moisture probe (Imko GmbH TRIME‐TDR) in July 2013. Five soil cores of 1.5 × 5 cm were taken from *c*. 10 cm below the surface using a soil auger during the same time period. The soil was homogenised, dried at 60°C for 96 hr and finely milled before analysis. Soil pH was measured from 10 g of the dry, milled soil, with a 1:5 ratio of soil to distilled water, using a pH probe (Dr.Meter^®^ 0.01pH Resolution pH Meter). A further 5 g was tested for total carbon and total nitrogen content at the Forest Research Centre for Ecosystems, Society, and Biosecurity (Farnham, UK), using a combustion method with a Carlo Erba CN analyser (Flash 1112 series).

### 2.3 Vegetation survey

A vegetation survey was conducted in July 2013 to determine the above‐ground plant community composition. A 0.5 × 0.5 m quadrat was placed randomly on the ground at each habitat patch and photographed using a Nikon camera. The photographs were analysed according to the methods for sampling vascular plants suggested by Peet, Wenthworth, and White (1998), using cover classes to describe the percentage cover of each species: Class 1 (0%–1%), Class 2 (1%–2%), Class 3 (2%–5%), Class 4 (5%–10%), Class 5 (10%–25%), Class 6 (25%–50%), Class 7 (50%–75%), Class 8 (75%–95%) and Class 9 (95%–99%). The mid‐points of the cover classes were used for further analysis of vegetation cover (e.g. if a plant species had a cover in Class 2 [1%–2%], we gave it the value 1.5%). All vascular plants, except for graminoids, were identified to species level with nomenclature following Kristinsson and Sigurdsson (2010), while the percentage cover of grasses, mosses, lichens and litter was also noted.

### 2.4 Invertebrate sampling

Above‐ground terrestrial invertebrate communities were sampled using pitfall traps in August 2012 and July 2013. Five pitfall traps were established at each habitat patch in a 1 m^2^ area, with one trap at each of the four corners and one in the centre (for a more comprehensive trapping area). White plastic cups of 7 cm diameter and 8.5 cm depth were filled with 10 ml of ethylene glycol and 30 ml of stream water, and left for 48 hr before collection (after Woodcock, 2007). Ethylene glycol was used for pitfall trapping as it prevents the escape of invertebrates from the traps and acts as a preservative. During collection, samples from the five traps at each habitat patch were combined into a 250‐μm sieve and stored in 70% ethanol. Note that a fine mesh size was used to prevent loss of small organisms such as mites and springtails. Terrestrial invertebrates were identified to species level where possible. We will refer to all taxa as species from henceforth.

### 2.5 Statistical analysis

All statistical analyses were carried out in r 3.2.0. Pearson correlations were used to test for relationships between temperature and pH, moisture, total carbon and total nitrogen (using the *cor* function in the *stats* package). Pairwise distances between sites were computed from the GPS coordinates, using the *earth.dist* function in the *fossil* package. A Mantel test was then used to test for spatial dependency of the temperature gradient, by comparing pairwise temperature difference to the pairwise distance between sites, using the *mantel* function in the *vegan* package.

The α‐diversity of the plant and invertebrate assemblages was assessed as Shannon–Weiner diversity (*H′*, using the *diversity* function in the *vegan* package), species richness (*S*) and Pielou's evenness (*J′* = *H′*/ln[*S*]) at each site. To avoid assumptions about linearity, the effect of temperature on the α‐diversity of invertebrates and plants in July 2013 was explored with generalised additive models (GAM: *gam* function in the *mgcv* package, with *k = *5 and *bs* = “*cr*”, after Keele, 2008). Analyses incorporating the main and interactive effects of temperature and year on the α‐diversity of invertebrates are presented in Supporting Information. Species turnover of the vegetation and invertebrate assemblages was quantified as the Sørensen similarity between pairwise combinations of all sites: β = (2*S*_*T*_/(*S*_1_ + *S*_2_)) − 1, where *S*_1_ and *S*_2_ are the species richness for sites 1 and 2, respectively, and *S*_*T*_ is the total species richness of the two sites. Note that greater similarity corresponds to lower species turnover between sites. A Mantel test was used to analyse the relationship between community similarity and pairwise temperature difference between sites for both plants and invertebrates.

Invertebrate population abundance in each habitat patch was estimated as the number of individuals of a species found in the five traps, with abundance at the site level taken as the mean value of the three habitat patches at each site. The total abundance of the invertebrate community was the sum of all population abundances per site. Individual body masses were estimated by measuring one linear dimension (body length) and converting to dry weight (mg) using length–weight relationships (Table S2, Figure S1). All individuals were measured apart from mites and springtails, where 30 individuals were measured for each habitat patch. From these data, the mean body mass of each species and the abundance‐weighted arithmetic mean body mass (henceforth “mean body mass”) of the invertebrate community were estimated for each site. Invertebrate population biomass was estimated as the abundance of each species per habitat patch multiplied by its mean body mass, while the total biomass of the invertebrate community was the sum of all population biomasses.

The effect of temperature on the percentage cover of the plant community and the mean body mass, total abundance and total biomass of the invertebrate community in July 2013 were explored with GAM. Analyses incorporating the main and interactive effects of temperature and year on the mean body mass, total abundance and total biomass of the invertebrate community are presented in Supporting Information. For the population‐level analyses, we explored the effect of temperature on the percentage cover of vegetation taxa and the mean body mass, abundance and biomass of each invertebrate species with GAM. We excluded species that were found at fewer than 10 sites to avoid biases associated with small sample size. All abundance, mean body mass and biomass data were transformed with log_10_(*x*), or log_10_(*x *+* *1) if zeros were present, to meet the assumptions of normality and homogeneity of variance and given our a priori expectation that these metrics should approximate a log‐normal distribution (Jonsson, Cohen, & Carpenter, 2005). The false discovery rate was used to avoid issues with multiple comparisons in the population‐level analyses (Benjamini & Hochberg, 1995), using the *p.adjust* function in the *stats* package, where *n* was the number of population‐level comparisons for percentage cover of vegetation taxa or the mean body mass, abundance or biomass of invertebrate species.

To determine whether the plant community may be indirectly mediating changes in the invertebrate community, we repeated the GAM analyses of invertebrate community metrics with plant community metrics included as covariates with temperature. Here, either the species richness, evenness, diversity, mean body mass, total abundance or total biomass of invertebrates was the response variable, and either the species richness, evenness, diversity or percentage cover of plants was a covariate with soil temperature. Note that structural equation modelling could not be implemented as a tool to determine relationships between our measured variables because of small sample size (i.e. 32 independent sites; Kline, 2015) and poor fittings between models and our data (i.e. root mean square error of approximation >0.2 in all analyses; MacCallum, Browne, & Sugawara, 1996).

All models were tested for spatial autocorrelation using the *spline.correlog* and *mantel.test* functions in the *ncf* package, with *x* coordinates corresponding to distance between sites along a North–South axis, *y* coordinates corresponding to distance between sites along a West–East axis, and model residuals as the observations at each site. There was little evidence for spatial autocorrelation of residuals in any analysis (Figures S2–S4), so no spatial structure was incorporated into any of the models.

## 3 RESULTS

### 3.1 Environmental variables

No significant correlation was found between temperature and any of the environmental variables measured in July 2013: pH (Pearson correlation: *r *=* *−.20, *t*_28_ *= *−1.085, *p *=* *.287), moisture (Pearson correlation: *r *=* *−.03, *t*_30_ = −0.166, *p *=* *.869), total carbon (Pearson correlation: *r *=* *−.32, *t*_30_ = −1.860, *p *=* *.073) or total nitrogen (Pearson correlation: *r *= −.28, *t*_30_ = ‐1.628, *p *=* *.114). Temperature was also not correlated with spatial distance between sites in either 2012 (Mantel test: *r *=* *.07, *p *=* *.256) or 2013 (Mantel test: *r *=* *.01, *p *=* *.373).

### 3.2 Vegetation

**Figure 2:**
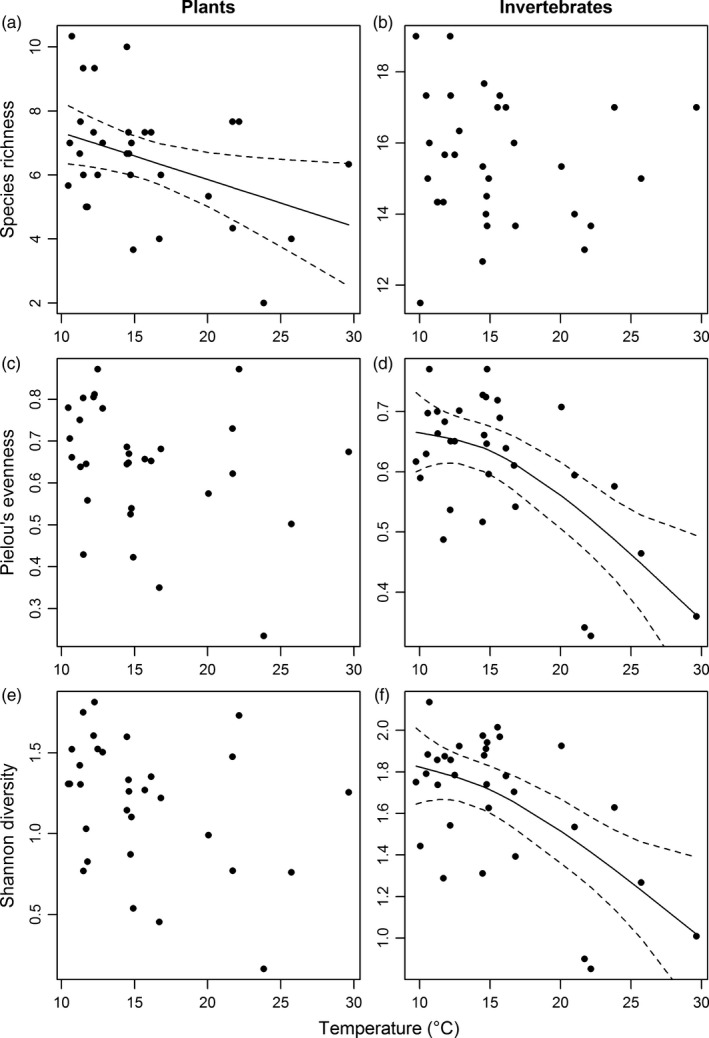
Relationships between soil temperature and various metrics of α‐diversity in July 2013: (a) plant species richness; (b) invertebrate species richness; (c) Pielou's evenness for plants; (d) Pielou's evenness for invertebrates; (e) Shannon diversity for plants; (f) Shannon diversity for invertebrates. Solid and dashed lines are the predicted fitting and 95% confidence intervals, respectively, from significant GAM models

**Figure 3:**
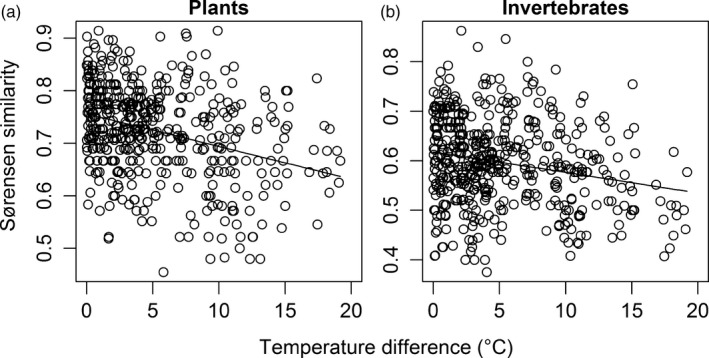
Declining Sørensen similarity in community composition with increasing pairwise temperature difference between sites in July 2013: (a) plants (*y *=* *0.756−0.006*x*); (b) invertebrates (*y *=* *0.618−0.004*x*)

There was a significant decrease in plant species richness with increasing soil temperature (GAM: *r* ^2^ = .12, *F *=* *5.183, *p *=* *.030; Figure 2a), partially supporting our first hypothesis. There was, however, no significant relationship between soil temperature and the Pielou's evenness (GAM: *r* ^2^ = .04, *F *=* *2.099, *p *=* *.155; Figure 2c) or Shannon diversity (GAM: *r* ^2^ = .07, *F *=* *3.294, *p *=* *.080; Figure 2e) of the plant community at Hengill. There was a significant decrease in community similarity (i.e. increased species turnover) with increasing pairwise temperature difference between sites (Mantel test: *r *=* *.327, *p *=* *.006; Figure 3a), supporting our second hypothesis.

In contrast to our fourth hypothesis, there was no significant effect of temperature on the percentage cover of plants at the community level (GAM: *r* ^2^ < .01, *F *=* *0.099, *p *=* *.755). There was limited support for our fourth hypothesis at the population level, with a significant decrease in the percentage cover of the Alpine bistort *Persicaria vivipara* with increasing soil temperature (Table S3; Figure S5a). There was an increase in the percentage cover of the willowherb *Epilobium* sp. from 10°C to 20°C and a decrease in percentage cover at higher soil temperatures (Table S3; Figure S5b). There was also a significant increase in the percentage cover of lichens, the thymeleaf speedwell *Veronica serpyllifolia* and the marsh violet *Viola palustris* with increasing soil temperature (Table S3; Figure S5c–e), in direct contrast to our fourth hypothesis. There were no significant effects of temperature on the percentage cover of mosses, grasses, litter or any other plant species (Table S3).

### 3.3 Invertebrate community

There was no effect of temperature on invertebrate species richness (GAM: *r* ^2^ < .01, *F *=* *0.133, *p *=* *.718; Figure 2b), but there was a significant reduction in Pielou's evenness (GAM: *r* ^2^ = .38, *F *=* *9.420, *p *<* *.001; Figure 2d) and Shannon diversity (GAM: *r* ^2^ = .34, *F *=* *7.984, *p *=* *.002; Figure 2f) of the invertebrate community as soil temperature increased, partially supporting our first hypothesis. There was also a significant decrease in community similarity (i.e. increased species turnover) with increasing pairwise temperature difference between sites (Mantel *r *=* *.218, *p *=* *.022; Figure 3b), supporting our second hypothesis. Similar results were found in the August 2012 pilot study (Figure S6).

**Figure 4:**
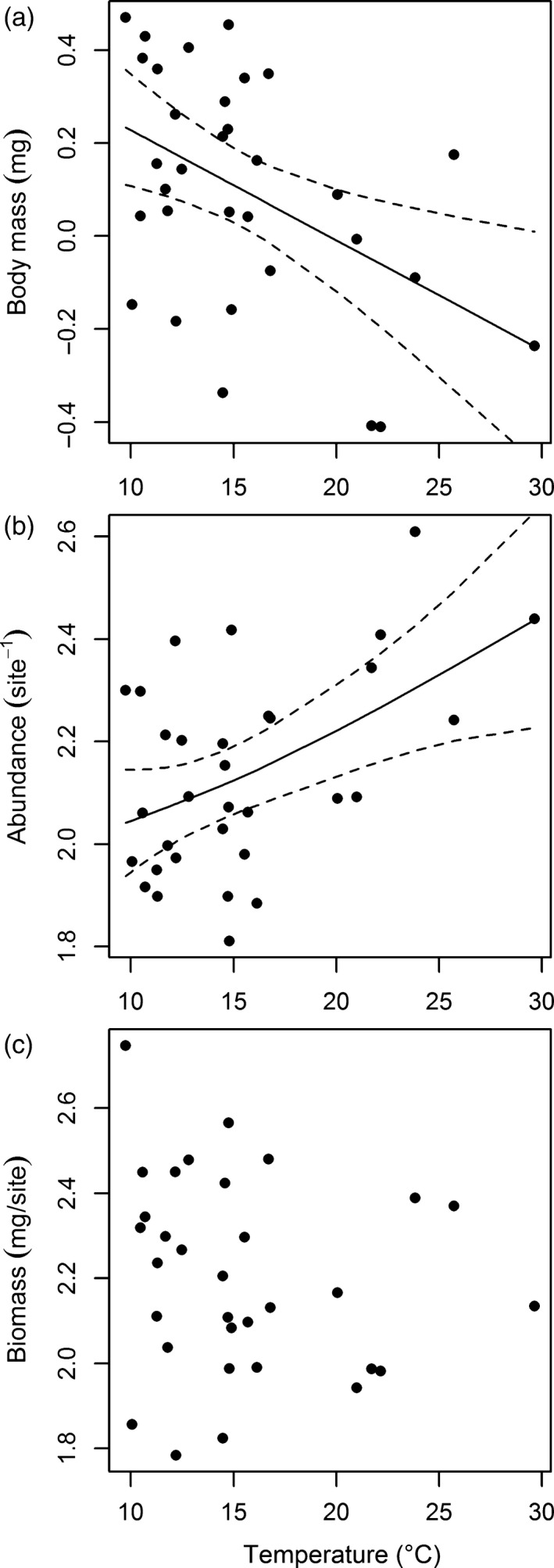
Relationships between soil temperature and (a) mean body mass; (b) total abundance; (c) total biomass of the invertebrate community in July 2013. Solid and dashed lines are the predicted fitting and 95% confidence intervals, respectively, from significant GAM models

There was a significant decrease in the mean body mass of the invertebrate community with increasing soil temperature (GAM: *r* ^2^ = .19, *F *=* *8.273, *p *=* *.007, Figure 4a), in line with our third hypothesis. A similar response was detected in August 2012 (Figure S7a). There was limited support for our third hypothesis at the population level, with a significant decrease in the mean body mass of parasitoid wasps (Platygastridae spp.; Table S4; Figure S8a). However, there was an increase in the mean body mass of springtails (Collembola spp.) and true bug nymphs (Hemiptera spp.) from 10°C to 15°C before declining at higher temperatures (Table S4; Figure S8b,c) and no significant effects on the remaining species (Table S4).

In contrast to our fourth hypothesis, there was a significant increase in the total abundance of terrestrial invertebrates with increasing soil temperature (GAM: *r* ^2^ = .22, *F *=* *5.879, *p *=* *.014, Figure 4b). A similar response was detected in August 2012 (Figure S7b). There were contrasting effects of temperature on population‐level abundances, partially supporting our fourth hypothesis. Here, adult flies (Diptera spp.), the harvestman *Mitopus morio*, the wolf spider *Pardosa palustris* and the carabid beetle *Patrobus septentrionis* decreased in abundance with increasing soil temperature (Table S4; Figure S9a–d). Conversely, earthworms (Annelida spp.), springtails, and the carabid beetles *Pterostichus diligens* and *Pterostichus nigrita* increased in abundance with increasing soil temperature (Table S4; Figure S9e–h). There were no significant changes in population abundance of the remaining invertebrate species across the soil temperature gradient (Table S4).

In contrast to our fourth hypothesis, there was no effect of temperature on the total biomass of terrestrial invertebrates with increasing soil temperature in July 2013 (GAM: *r* ^2^ < .01, *F *=* *0.731, *p *=* *.402, Figure 4c) or August 2012 (Figure S7c). There were contrasting effects of temperature on population‐level biomasses, partially supporting our fourth hypothesis. Here, *P. palustris* and *P. septentrionis* decreased in biomass with increasing soil temperature (Table S4; Figure S10a,b), while there was an increase in the biomass of the wolf spider *Pirata piraticus* and both *P. diligens* and *P*. *nigrita* with increasing soil temperature (Table S4; Figure S10c–e). There were no significant changes in biomass of the remaining invertebrate species across the soil temperature gradient (Table S4).

### 3.4 Plant‐mediated effects on invertebrates

There was a significant effect of Pielou's evenness of the plant community on both Pielou's evenness (GAM: *r* ^2^ = .45, *F *=* *5.968, *p *=* *.021) and Shannon diversity (GAM: *r* ^2^ = .41, *F *=* *5.024, *p *=* *.033) of the invertebrate community when it was included as a covariate with soil temperature in the analysis. There were no other significant effects of plant community metrics on invertebrate community metrics when the former were included as covariates in the analyses of soil temperature effects on the latter (Table S5).

## 4 DISCUSSION

### 4.1 Diversity and percentage cover of plants

There were fewer plant species found as soil temperature increased (Figure 2a), which likely reflects the dearth of species with higher thermal optima in the regional species pool (Kristinsson & Sigurdsson, 2010). Similar effects have been found along elevational gradients, where invasion of warm‐adapted species could not offset the loss of native plant species at higher temperatures (De Sassi, Lewis, & Tylianakis, 2012). The early developmental stages of plants are likely to be more sensitive to environmental constraints, with warming shown to decrease the species richness of emerging seedlings (Lloret, Penuelas, & Estiarte, 2004). This may be particularly true for *P*. *vivipara*, which was rarely found at warmer soil temperatures (Figure S5a) and is most susceptible to heat stress following germination (Marcante, Erschbamer, Buchner, & Neuner, 2014), with a lower thermal tolerance than many of its alpine competitors, such as *Epilobium* sp. (Schwienbacher, Navarro‐Cano, Neuner, & Erschbamer, 2012). It was perhaps surprising that there was a greater percentage cover of *V*. *palustris* in warmer soil (Figure S5e) given that it typically experiences reduced emergence of seedlings at higher temperatures (Klanderud, Meineri, Töpper, Michel, & Vandvik, 2017), although this may be driven by its increased performance at higher temperatures after loss of dominant competitors (Olsen, Töpper, Skarpaas, Vandvik, & Klanderud, 2016).

While competition for space and soil nutrients should have been lower as a result of fewer species, there were no changes in evenness and thus dominance of plants across the temperature gradient. However, large‐scale patterns in declining diversity are often masked by variation at regional or local scales (Walker et al., 2006). There was also an increased turnover of plant species as soil temperature increased (Figure 3a), which is predicted for many plant communities under a warming climate (Thuiller, 2004) and is most likely related to temperature niche separation amongst species (Bertrand et al., 2011). Thus, plant species with a low thermal optimum (e.g. *P*. *vivipara*) are likely to be excluded in the warmest sites, where they may be replaced by more warm‐adapted plants (e.g. *Epilobium* sp.). This phenomenon has been demonstrated in highland areas of France in response to just a 1°C increase in temperature over recent decades (Bertrand et al., 2011). Note that increases in the percentage cover of lichens and *V*. *serpyllifolia* with increasing soil temperature were driven by a single influential data point in each case (Cook's distance of 9.2 and 9.8 respectively). As such, these trends are unlikely to be representative of general warming impacts on these taxa, especially given the widespread negative effect of temperature on terrestrial lichens (Lang et al., 2012; Walker et al., 2006).

### 4.2 Invertebrate diversity

Invertebrate species evenness, although not richness, decreased in the warmer sites, leading to an overall decline in Shannon diversity (Figure 2). This suggests that warming had a marked effect on community composition, with the wolf spider *P. piraticus* and beetles from the genus *Pterostichus* dominating the warmer habitat patches (Figure S10). These species have a high thermal tolerance (Berthe et al., 2015; Nørgaard, 1951) and may be able to disperse to their optimum temperature due to their mobility (e.g. Juliano, 1983). These changes in evenness are supported by evidence for a reduction in invertebrate diversity following experimental warming due to shifts in species dominance (Berthe et al., 2015; Villalpando, Williams, & Norby, 2008). There was also high invertebrate species turnover along the temperature gradient, as seen in thermally influenced marine benthic communities (Hillebrand et al., 2010). Here, the harvestman *M*. *morio* and the beetle *P*. *septentrionis* in particular were absent from sites >17°C, where they were replaced by the *Pterostichus* beetles (see Figure S9). Similar effects have also been observed in the freshwater streams at Hengill, where community composition changed dramatically in the warmer streams, with a few dominant species reducing the evenness of the invertebrate community (Woodward, Dybkjaer, et al., 2010).

Changes in invertebrate community composition may also have been indirectly mediated by the vegetation, rather than just direct effects of temperature on invertebrate physiology and behaviour (De Sassi et al., 2012). For example, changes in the evenness of the plant community across the soil temperature gradient were shown to independently contribute to the reduction in evenness and diversity of the invertebrate community, as suggested by the stronger temperature effect on invertebrate community composition after taking plant evenness into account (Table S5). Changes in plant community composition have previously been shown to alter the abundance and species richness of herbivorous invertebrates (Zhou et al., 2015), which might help to explain the changes we observed in the abundance of springtails in our study and even some of the predatory beetles whose diet frequently consists of plant material (Dawson, 1965). Similarly, less diverse vegetation provides fewer refugia for herbivorous invertebrates, resulting in greater top–down control by predators (Sanders, Nickel, Grützner, & Platner, 2008). For example, the predatory *Pterostichus* beetles may be aggregating at warmer patches due to the availability of their springtail prey there (Figure S9; Dawson, 1965; Mundy, Allen‐Williams, Underwood, & Warrington, 2000). Indeed, declining plant species richness has stronger negative effects on progressively lower trophic levels in above‐ground food webs (Scherber et al., 2010), which could help to explain the loss of invertebrate diversity seen here (Figure 2f). Given that the sampling period for the study took place under the near‐equilibrium conditions of late summer, and not during the community assembly phase, it would be interesting to examine plant and invertebrate species composition as a function of time following the spring snowmelt and early plant growth, right through to winter snowfall. Transient differences in plant community assembly as a result of soil temperature decrease could result in legacy effects on the associated invertebrate community (Bale et al., 2002; Dollery et al., 2006; Liu, Reich, Li, & Sun, 2011), especially considering how plant identity may play a more important role in insect herbivore colonisation than temperature alone (Andrew & Hughes, 2007).

### 4.3 Invertebrate body mass

The mean body mass of the invertebrate community decreased with increasing soil temperature (Figure 4a), in line with our third hypothesis and echoing recent meta‐analyses on freshwater communities (Daufresne et al., 2009) and oceanic phytoplankton (Moran, Lopez‐Urrutia, Calvo‐Diaz, & Li, 2010). This suggests that temperature effects on the behaviour and physiology of invertebrates were strong despite the relatively interconnected nature of the warm and cold sites that we studied and indications that temperature effects are much weaker on the body size of terrestrial than aquatic organisms (Forster, Hirst, & Atkinson, 2012). The general lack of temperature effects on mean body mass at the population level (Table S4) suggests that this finding was driven by the observed species turnover in the invertebrate community (Figure 4b,c), with smaller species at higher soil temperatures rather than smaller individuals within each population in the community (except for parasitoid wasps; see Figure S8a). This finding is of particular note given the short distances between sites of different temperature in the Hengill system and indicates a clear preferential use of warmer habitat by smaller species (e.g. springtails; see Figure S9f).

In contrast to the population‐level component of our third hypothesis, we only observed a decrease in the mean body mass of parasitoid wasps with increasing soil temperature. Furthermore, there was an increase in the mean body mass of springtails and true bug nymphs from 10 to 15°C, before their body mass decreased at higher temperatures (Figure S8b,c), with no effects on the remaining invertebrate species. Variable population‐level responses to warming have been regularly reported (Gardner et al., 2011; Watt, Mitchell, & Salewski, 2010), including idiosyncratic trends among insects (Mousseau, 1997; Shelomi, 2012; Walters & Hassall, 2006) and a decrease in the body size of some Carabidae at cooler, high latitudes (Ikeda, Tsuchiya, Nagata, Ito, & Sota, 2012). It is possible that population‐level variability in temperature–size responses are a consequence of developmental and functional constraints on body mass and individual plasticity (Angilletta, Steury, & Sears, 2004; Forster, Hirst, & Woodward, 2011; Moya‐Larano et al., 2012). Voltinism (i.e. the number of generations per year) may also help to explain the contrasting temperature–size responses of terrestrial species, with long‐lived univoltine organisms able to achieve large size over a single growing season, while multivoltine organisms often maximise their fitness by maturing earlier at smaller size (Horne, Hirst, & Atkinson, 2015). Contradictions of temperature–size rules have also been observed in the Hengill streams, at both the community and population levels, potentially as a consequence of temperature‐mediated increase in resource availability, or enhanced rates of growth and reproduction (e.g. Adams et al., 2013; O'Gorman et al., 2012). Clearly, more research is needed on this topic to determine the idiosyncratic differences in temperature–size responses for various taxonomic groups.

### 4.4 Invertebrate abundance and biomass

There was a positive effect of temperature on the total abundance of invertebrates (Figure 4b), in contrast to our fourth hypothesis. Similar increases in the abundance of soil invertebrates with warming are also predicted for Arctic areas in response to changes in vegetation type (Nielsen & Wall, 2013). Shifts in plant community composition, such as those observed here (Figure 3a), have also been shown to mediate a greater abundance of herbivores at higher temperatures (De Sassi et al., 2012). While there were more invertebrates at the warmer sites, the concurrent reduction in mean body size ensured that overall community biomass was unaffected by temperature (Figure 4c). Resource usage by the consumer community thus seemingly kept pace with resource provisioning, with no overall change in percentage cover of the vegetation community as soil temperature increased. Faster rates of vegetation regeneration at higher temperatures (Nishar et al., 2017) may play a key role in meeting the higher metabolic demands of herbivores and subsequently their predators. Arctic and subarctic organisms are also predicted to exhibit relatively small absolute shifts in metabolic rate compared to those at lower latitudes due to their cooler baseline temperatures and the exponential relationship between metabolism and temperature (Dillon, Wang, & Huey, 2010).

There were contrasting population‐level responses to temperature (Figures S9 and S10), which is most likely due to variation in the thermal tolerances of individual species. For example, *M*. *morio* and *P. septentrionis* are most commonly associated with cold alpine environments (Bakken, 1985; Hein, Pape, Finch, & Löffler, 2014), reflecting their occurrence at only the coldest sites here (Figure S9). Indeed, *M*. *morio* prefers cool, moist habitats because it is susceptible to desiccation at higher temperatures (Hein et al., 2014). In contrast, *Pterostichus* beetles have been shown to thrive after experimental warming (Berthe et al., 2015), mirroring the increased abundance and biomass of *P. diligens* and *P. nigrita* here (Figures S9 and S10). Contrasting effects of temperature have also been shown on invertebrate populations in German forests (Müller‐Kroehling, Jantsch, Fischer, & Fischer, 2014).

The abundance and biomass of species in riparian ecosystems may also be influenced by aquatic subsidies. For example, adult insect emergence can alter terrestrial riparian communities, with the diets of dominant beetle and spider species in some studies shown to consist largely of aquatic insects (Paetzold, Schubert, & Tockner, 2005). Greater insect emergence has been demonstrated from warmer waters (Greig et al., 2012), including at our study system (Hannesdottir et al., 2013), which may provide supplementary resources to predatory invertebrates on the surrounding banks, leading to their aggregation at warmer soil patches. Due to the natal site fidelity observed for many insect species (e.g. Krosch, Baker, Mather, & Cranston, 2011), terrestrial invertebrates may return to these warmer and resource‐rich patches across generations, resulting in consistently higher abundances of terrestrial invertebrates at these sites. This trend may be amplified by the effect of temperature on voltinism, with warming shown to increase the number of generations per year in European butterflies and moths (Altermatt, 2010). Thus, temperature effects on stream subsidies to the terrestrial invertebrate community should be explored and quantified to better understand their contribution to the observed patterns.

## 5 CONCLUSIONS

Warming is widely predicted to negatively affect the diversity, body size and biomass of natural communities (Daufresne et al., 2009; Millennium Ecosystem Assessment 2005; Pimm, 2009). We found support for reductions in the diversity of plant and ground‐dwelling invertebrate communities at higher temperatures. We observed a smaller mean body size of invertebrates at the community level in warmer environments, although generally not at the population level, offering mixed support for temperature–size rules. The surprising increase in total abundance of the invertebrate community at warmer sites was offset by their smaller size, resulting in no net change in invertebrate biomass across the temperature gradient. This suggests that consumers had sufficient resources, despite their higher energy demands at warmer temperatures. Inconsistent population‐level effects of temperature on the percentage cover of vegetation and the abundance and biomass of invertebrate species in the system highlight the need for community‐level research to understand the ecosystem‐level consequences of these context‐dependent effects. An analysis of changes in trophic network structure in response to temperature would be a logical next step, to determine how the observed changes in plant and invertebrate community structure might alter the flow of energy through the food web. Understanding the underlying mechanisms of population‐ and community‐level change will help to identify the emergent properties of ecosystems and mitigate the consequences of climate change.

## AUTHORS’ CONTRIBUTIONS

O.B.M. and E.J.O. conceived the ideas and designed methodology; O.B.M., E.J.O. and B.M. collected the data; S.I.R., E.J.O., and B.M. analysed the data; S.I.R. and E.J.O. led the writing of the manuscript. All authors contributed critically to the drafts and gave final approval for publication.

## DATA ACCESSIBILITY

All data is archived with the NERC Environmental Information Data Centre https://doi.org/10.5285/0f074839-1630-4ccd-aa63-84d0da16b28a (O'Gorman, Robinson, McLaughlin, & Marteinsdóttir, 2017).
