# Supplementary material for: Soil temperature effects on the structure and diversity of plant and invertebrate communities in a natural warming experiment
Source: J Anim Ecol. 2018 Feb 13;87(3):634–46. doi: 10.1111/1365-2656.12798 (PMC6849623; doi:10.1111/1365-2656.12798)
Supplement: Supplementary file 1 [file JANE-87-634-s001.doc]

**Supporting Information: Soil temperature effects on the structure and diversity of plant and invertebrate communities in a natural warming experiment**

Sinikka I. Robinson^1^, Órla B. McLaughlin^2^, Bryndís Marteinsdóttir^3^, and Eoin J. O’Gorman^1^

*^1^ Department of Life Sciences, Imperial College London, Silwood Park Campus, Buckhurst Road, Ascot, Berkshire SL5 7PY, UK.*

*^2^ Agroécologie, AgroSup Dijon, INRA, Univ. Bourgogne Franche-Comté, F-21000 Dijon, France.*

*^3^ Institute of Life and Environmental Sciences, University of Iceland, Askja - Sturlugata 7, 101 Reykjavík, Iceland.*

**Table S1. Mean values of environmental variables measured at the 32 terrestrial sites in the Hengill valley.** Site codes refer to the left (L) and right (R) banks of the stream numbers shown in Figure 1. The temperature (temp. in °C), total carbon (TC in g kg^-1^), total nitrogen (TN in g kg^-1^), percentage moisture, and pH of the soil at each site are also provided. All data were collected in July 2013, apart from spot temperature measurements taken in August 2012. Mean and standard deviation (SD) of soil temperatures in July 2013 were estimated from measurements recorded every ten minutes during a 48-hour period (see *Methods*).

| **Site** | **Spot temp. 2012** | **Mean temp. 2013** | **SD temp. 2013** | **TC** | **TN** | **Moisture** | **pH** |
| --- | --- | --- | --- | --- | --- | --- | --- |
| 1L | 8.6 | 14.5 | 1.6 | 5.99 | 0.45 | 60.5 | 5.8 |
| 1R | 7.9 | 12.3 | 1.6 | 3.13 | 0.24 | 61.7 | 6.0 |
| 2L | 13.0 | 23.8 | 9.7 | 6.59 | 0.51 | 61.7 | 4.7 |
| 2R | 7.8 | 14.9 | 2.1 | 6.70 | 0.53 | 49.6 | 4.8 |
| 3L | 18.5 | 25.7 | 2.0 | 2.18 | 0.14 | 88.6 | 4.7 |
| 3R | 18.0 | 21.7 | 1.3 | 3.10 | 0.19 | 83.6 | 5.1 |
| 4L | 14.4 | 16.8 | 4.8 | 1.63 | 0.10 | 54.7 | 5.7 |
| 4R | 15.4 | 20.1 | 4.2 | 2.20 | 0.12 | 56.2 | 6.1 |
| 5L | 9.7 | 14.6 | 2.0 | 7.41 | 0.38 | 79.8 | 5.4 |
| 5R | 10.1 | 16.7 | 2.3 | 3.58 | 0.25 | 76.7 | 5.3 |
| 6L | 9.8 | 16.1 | 2.2 | 7.41 | 0.51 | 69.0 | 5.8 |
| 6R | 9.4 | 15.7 | 2.6 | 8.81 | 0.65 | 72.0 | 5.9 |
| 7L | 8.7 | 14.5 | 2.0 | 6.73 | 0.56 | 78.2 | 5.3 |
| 7R | 5.9 | 12.8 | 2.0 | 12.84 | 0.89 | 82.1 | 5.0 |
| 8L | 8.5 | 14.5 | 2.0 | 9.19 | 0.63 | 74.4 | 5.4 |
| 8R | 9.0 | 14.6 | 2.2 | 6.56 | 0.46 | 78.4 | 5.6 |
| 9L | 8.7 | 14.7 | 2.2 | 5.83 | 0.41 | 68.8 | 6.5 |
| 9R | 8.1 | 14.8 | 1.6 | 8.98 | 0.54 | 63.6 | 5.4 |
| 10L | 3.9 | 10.5 | 2.4 | 4.14 | 0.29 | 76.4 | 5.8 |
| 10R | 4.1 | 10.7 | 1.7 | 6.17 | 0.31 | 69.5 | 7.1 |
| 11L | 5.3 | 12.2 | 1.6 | 9.27 | 0.47 | 58.9 | 6.6 |
| 11R | 4.2 | 11.8 | 2.6 | 6.36 | 0.39 | 82.8 | 5.8 |
| 12L | 7.8 | 11.3 | 1.5 | 4.59 | 0.31 | 74.7 | n/a |
| 12R | 7.4 | 12.5 | 1.5 | 2.51 | 0.16 | 55.8 | 5.8 |
| 13L | 3.9 | 10.6 | 2.2 | 3.67 | 0.23 | 58.9 | 5.6 |
| 13R | 5.1 | 11.7 | 2.2 | 2.88 | 0.20 | 81.7 | 5.2 |
| 14L | 5.8 | 11.3 | 3.5 | 2.24 | 0.13 | 55.7 | 6.0 |
| 14R | 4.4 | 11.5 | 2.3 | 2.09 | 0.11 | 49.2 | n/a |
| 15L | 17.8 | 29.6 | 6.4 | 0.89 | 0.05 | 53.0 | 6.3 |
| 15R | 13.8 | 22.2 | 3.9 | 0.86 | 0.04 | 59.1 | 6.1 |
| 16L | 5.6 | 11.5 | 3.4 | 4.22 | 0.32 | 49.1 | 5.4 |
| 16R | 13.9 | 21.7 | 1.6 | 3.47 | 0.22 | 55.6 | 5.9 |

**Table S2. Length-weight (L-W) relationships for estimating the body mass of terrestrial invertebrate sampled in July 2013.** The taxa and group columns refer to the taxa identified in this study and the taxonomic group that the L-W relationship was derived for, respectively. The L‑W relationships all take the form log(*y*) = *a* + *b* * log(*x*), where *x* is body length in mm, *y* is dry mass in mg, and the values of *a* and *b* are provided in the table. The base column indicates whether the logarithms of *x* and *y* were calculated to the base 10 or *e* (*i*.*e*. the exponential base). The *r*^2^ value for each L-W relationship and reference to the study that it was extracted from are also provided, with "this study" referring to L-W relationships that were calculated specifically from invertebrates collected at Hengill in August 2012 (see Fig. S1).

| **Taxa** | **Group** | ***a*** | ***b*** | **Base** | ***r*^2^** | **Reference** |
| --- | --- | --- | --- | --- | --- | --- |
| Acari spp. | Order Acari | -1.23 | 2.33 | 10 | 0.73 | McLaughlin *et al.* (2010) |
| Annelida spp. | Order Oligochaeta | -1.69 | 1.66 | 10 | 0.50 | McLaughlin *et al.* (2010) |
| Aphididae spp. | Family Aphididae | -1.37 | 0.99 | 10 | 0.24 | McLaughlin *et al.* (2010) |
| *Aphodius lapponum* | Order Coleoptera | -1.7912 | 2.7877 | 10 | 0.89 | This study |
| *Arctorthezia cataphracta* | Order Hemiptera | -1.39 | 1.77 | 10 | 0.72 | McLaughlin *et al.* (2010) |
| *Arctosa alpigena* | *Arctosa alpigena* | -1.8100 | 3.0685 | 10 | 0.97 | This study |
| *Arctosa leopardus* | *Arctosa alpigena* | -1.8100 | 3.0685 | 10 | 0.97 | This study |
| *Arctosa lutetiana* | *Arctosa lutetiana* | -1.8844 | 3.1569 | 10 | 0.88 | This study |
| *Arion* spp. | *Arion* spp. | -2.2674 | 2.8740 | 10 | 0.82 | This study |
| *Bembidion bipunctatum* | Family Carabidae | -1.8344 | 2.8254 | 10 | 0.91 | This study |
| *Bembidion punctulatum* | Family Carabidae | -1.8344 | 2.8254 | 10 | 0.91 | This study |
| *Bombus lucorum* | Order Hymenoptera | -4.276 | 2.383 | *e* | 0.92 | Gruner (2003) |
| *Bombus terrestris* | Order Hymenoptera | -4.276 | 2.383 | *e* | 0.92 | Gruner (2003) |
| *Calathus ambiguus* | Family Carabidae | -1.8344 | 2.8254 | 10 | 0.91 | This study |
| *Calathus melanocephalus* | Family Carabidae | -1.8344 | 2.8254 | 10 | 0.91 | This study |
| *Cerapteryx graminis* | Family Noctuidae | -1.6839 | 2.3705 | 10 | 0.72 | This study |
| *Cerapteryx graminis* larva | Order Lepidoptera larvae | -3.607 | 1.769 | *e* | 0.68 | Gruner (2003) |
| *Cercyon analis* | Order Coleoptera | -1.7912 | 2.7877 | 10 | 0.89 | This study |
| Chironomidae spp. | Order Diptera | -4.18 | 2.573 | *e* | 0.85 | Gruner (2003) |
| Chironomidae spp. larva | Order Diptera larvae | -2.28 | 2.22 | 10 | 0.68 | McLaughlin *et al.* (2010) |
| Chironomidae spp. pupa | Order Diptera larvae | -2.28 | 2.22 | 10 | 0.68 | McLaughlin *et al.* (2010) |
| *Coccinella undecimpunctata* | Family Coccinellidae | -2.625 | 2.665 | *e* | 0.65 | Gruner (2003) |
| Coleoptera spp. larva | Order Coleoptera larvae | -1.43 | 1.33 | 10 | 0.43 | McLaughlin *et al.* (2010) |
| Collembola spp. | Order Collembola | -5.177 | 2.809 | *e* | 0.92 | Gruner (2003) |
| *Cytilus sericeus* | Order Coleoptera | -1.7912 | 2.7877 | 10 | 0.89 | This study |
| *Deroceras* spp. | *Deroceras* spp. | -1.7941 | 2.1108 | 10 | 0.76 | This study |
| Diptera spp. | Order Diptera | -4.18 | 2.573 | *e* | 0.85 | Gruner (2003) |
| Diptera spp. larva | Order Diptera larvae | -2.28 | 2.22 | 10 | 0.68 | McLaughlin *et al.* (2010) |
| Diptera spp. pupa | Order Diptera larvae | -2.28 | 2.22 | 10 | 0.68 | McLaughlin *et al.* (2010) |
| *Eana osseana* | Family Noctuidae | -1.6839 | 2.3705 | 10 | 0.72 | This study |
| Gastropod spp. | Order Gastropoda | -2.2704 | 2.6784 | 10 | 0.70 | This study |
| Geometrid spp. | Family Noctuidae | -1.6839 | 2.3705 | 10 | 0.72 | This study |
| Gonatium spp. | Family Linyphiidae | -1.1822 | 1.8992 | 10 | 0.54 | This study |
| Hemiptera spp. | Order Hemiptera | -1.39 | 1.77 | 10 | 0.72 | McLaughlin *et al.* (2010) |
| Hemiptera spp. nymph | Order Hemiptera | -1.39 | 1.77 | 10 | 0.72 | McLaughlin *et al.* (2010) |
| Hydrophilidae spp. | Order Coleoptera | -1.7912 | 2.7877 | 10 | 0.89 | This study |
| Hymenoptera spp. | Order Hymenoptera | -4.276 | 2.383 | *e* | 0.92 | Gruner (2003) |
| *Hypnoidus riparius* | Family Elateridae | -1.78 | 2.74 | 10 | 0.66 | McLaughlin *et al.* (2010) |
| Ichneumonidae spp. | Order Hymenoptera | -4.276 | 2.383 | *e* | 0.92 | Gruner (2003) |
| Isopoda spp. | Order Isopoda | -4.185 | 2.77 | *e* | 0.97 | Gruner (2003) |
| Lepidoptera spp. chrysalis | Order Lepidoptera larvae | -3.607 | 1.769 | *e* | 0.68 | Gruner (2003) |
| Lepidoptera spp. larva | Order Lepidoptera larvae | -3.607 | 1.769 | *e* | 0.68 | Gruner (2003) |
| Limnephilidae spp. | Family Noctuidea | -1.6839 | 2.3705 | 10 | 0.72 | This study |
| Linyphiidae spp. | Family Linyphiidae | -1.1822 | 1.8992 | 10 | 0.54 | This study |
| Lycosidae spp. juvenile | Family Lycosidae | -1.4457 | 2.6186 | 10 | 0.80 | This study |
| *Mitopus morio* | *Mitopus morio* | -1.1400 | 2.4170 | 10 | 0.75 | This study |
| *Nebria salina* | Family Carabidae | -1.8344 | 2.8254 | 10 | 0.91 | This study |
| *Nephus redtenbacheri* | Order Coleoptera | -1.7912 | 2.7877 | 10 | 0.89 | This study |
| *Notiophilus biguttatus* | Family Carabidae | -1.8344 | 2.8254 | 10 | 0.91 | This study |
| Oligochaeta spp. | Order Oligochaeta | -1.69 | 1.66 | 10 | 0.89 | McLaughlin *et al.* (2010) |
| *Otiorynchus nodosus* | Family Curculinoidea | -2.801 | 2.315 | *e* | 0.78 | Gruner (2003) |
| *Pardosa agrestis* | Family Lycosidae | -1.4457 | 2.6186 | 10 | 0.80 | This study |
| *Pardosa palustris* | Family Lycosidae | -1.4457 | 2.6186 | 10 | 0.80 | This study |
| *Pardosa prativaga* | Family Lycosidae | -1.4457 | 2.6186 | 10 | 0.80 | This study |
| *Patrobus septentrionis* | Family Carabidae | -1.8344 | 2.8254 | 10 | 0.91 | This study |
| *Phratora polaris* | Order Coleoptera | -1.7912 | 2.7877 | 10 | 0.89 | This study |
| *Piesma maculatum* | Order Heteroptera | -3.192 | 1.934 | *e* | 0.82 | Gruner (2003) |
| *Pirata hygrophila* | *Pirata hygrophila* | -1.3051 | 2.4412 | 10 | 0.75 | This study |
| *Pirata piraticus* | *Pirata piraticus* | -1.3886 | 2.5590 | 10 | 0.81 | This study |
| *Piratula knorri* | Family Lycosidae | -1.4457 | 2.6186 | 10 | 0.80 | This study |
| Platygastridae spp. | Order Hymenoptera | -4.276 | 2.383 | *e* | 0.92 | Gruner (2003) |
| Plecoptera spp. | Order Orthoptera | -4.017 | 2.720 | *e* | 0.92 | Gruner (2003) |
| *Porhomma montanum* | Family Linyphiidae | -1.1822 | 1.8992 | 10 | 0.54 | This study |
| *Potamophylax cingulatus* | Family Noctuidea | -1.6839 | 2.3705 | 10 | 0.72 | This study |
| *Pterostichus anthracinus* | *Pterostichus strenuus* | -1.3711 | 2.3807 | 10 | 0.72 | This study |
| *Pterostichus diligens* | *Pterostichus strenuus* | -1.3711 | 2.3807 | 10 | 0.72 | This study |
| *Pterostichus gracilis* | *Pterostichus strenuus* | -1.3711 | 2.3807 | 10 | 0.72 | This study |
| *Pterostichus melanarius* | *Pterostichus strenuus* | -1.3711 | 2.3807 | 10 | 0.72 | This study |
| *Pterostichus nigrita* | *Pterostichus strenuus* | -1.3711 | 2.3807 | 10 | 0.72 | This study |
| *Radix balthica* | Order Gastropoda | -2.2704 | 2.6784 | 10 | 0.70 | This study |
| Staphylinidae spp. | Family Staphylinidae | -1.99 | 2.09 | 10 | 0.51 | McLaughlin *et al.* (2010) |
| Staphylinidae spp. larva | Order Coleoptera larvae | -1.43 | 1.33 | 10 | 0.42 | McLaughlin *et al.* (2010) |
| Thysanoptera spp. | Order Thysanoptera | -5.18 | 1.89 | *e* | 0.54 | Wardaugh (2013) |
| Thysanoptera spp. nymph | Order Thysanoptera | -5.18 | 1.89 | *e* | 0.54 | Wardaugh (2013) |
| Tipulidae spp. | Order Diptera | -4.180 | 2.573 | *e* | 0.85 | Gruner (2003) |
| Tipulidae spp. larva | Order Diptera larvae | -2.28 | 2.22 | 10 | 0.68 | McLaughlin *et al.* (2010) |
| *Trechus fulvus* | Family Carabidae | -1.8344 | 2.8254 | 10 | 0.91 | This study |
| *Trechus obtusus* | Family Carabidae | -1.8344 | 2.8254 | 10 | 0.91 | This study |
| *Trechus subnotatus* | Family Carabidae | -1.8344 | 2.8254 | 10 | 0.91 | This study |

**References:**

Gruner DS (2003). Regressions of length and width to predict arthropod biomass in the Hawaiian Islands. *Pacific Science*, 57, 325-336.

McLaughlin OB, Jonsson T, Emmerson MC (2010). Temporal variability in predator-prey relationships of a forest floor food web. *Adv. Ecol. Res.*, 42, 171-264.

Wardhaugh CW (2013). Estimation of biomass from body length and width for tropical rainforest canopy invertebrates. *Aust. J. Entomol.*, 52, 291-298.

**Table S3.** *F*, *p*, and *r*^2^ values from the GAM analyses of percentage cover of plant species and other major vegetation groups sampled in July 2013 as a function of temperature. Analyses were only performed on taxa where the number of occurrences across all sites was > 10.

| **Species** | ***F* value** | ***p* value** | ***r*^2^** |
| --- | --- | --- | --- |
| Moss | 2.177 | 0.178 | 0.14 |
| Grasses | 1.859 | 0.236 | 0.12 |
| Lichens | 6.446 | 0.015 | 0.38 |
| Litter | 1.512 | 0.283 | 0.07 |
| *Alchemilla alpina* | 3.092 | 0.150 | 0.10 |
| *Alchemilla mollis* | 3.363 | 0.150 | 0.07 |
| *Cardaminopsis petraea* | 1.815 | 0.240 | 0.09 |
| *Carex sp.* | 0.722 | 0.520 | 0.02 |
| *Cerastium fontanum* | 2.525 | 0.150 | 0.19 |
| *Epilobium sp.* | 6.347 | 0.015 | 0.36 |
| *Equisetum sp.* | 1.650 | 0.269 | 0.02 |
| *Leontodon autumnalis* | 3.874 | 0.141 | 0.10 |
| *Persicaria vivipara* | 6.315 | 0.015 | 0.32 |
| *Rumex acetosa* | 1.027 | 0.441 | 0.04 |
| *Taraxacum sp.* | 0.171 | 0.683 | <0.01 |
| *Thymus praecox* | 5.252 | 0.088 | 0.12 |
| *Veronica serpyllifolia* | 10.67 | 0.001 | 0.54 |
| *Viola palustris* | 7.521 | 0.037 | 0.18 |

**Table S4.** *F*, *p*, and *r*^2^ values from the GAM analyses of the mean body mass, abundance, and biomass of invertebrate species sampled in July 2013 as a function of temperature. Analyses were only performed on taxa where the number of occurrences across all sites was > 10. Note that zeros were included in the estimation of abundance and biomass, where a zero indicates that a species is simply not present at a site, but not in the estimation of mean body mass, since an organism cannot have a body mass of zero. Thus, there are some NA values for mean body mass, reflecting insufficient data for GAM analysis.

|  | **Mean body mass** | | | **Total abundance** | | | **Total biomass** | | |
| --- | --- | --- | --- | --- | --- | --- | --- | --- | --- |
| **Species** | ***F* value** | ***p* value** | ***r*^2^** | ***F* value** | ***p* value** | ***r*^2^** | ***F* value** | ***p* value** | ***r*^2^** |
| Acari spp. | 0.965 | 0.566 | 0.05 | 1.626 | 0.316 | 0.03 | 0.672 | 0.568 | 0.03 |
| Annelida spp. | NA | NA | NA | 7.964 | 0.034 | 0.18 | 3.124 | 0.267 | 0.06 |
| Aphidiidae spp. | 0.013 | 0.979 | <0.01 | 1.417 | 0.343 | 0.08 | 2.832 | 0.283 | 0.06 |
| *Arctorthezia cataphracta* | 0.285 | 0.845 | <0.01 | 1.292 | 0.349 | 0.01 | 0.174 | 0.680 | <0.01 |
| *Arion* spp. | 0.006 | 0.979 | <0.01 | 0.069 | 0.916 | <0.01 | 0.987 | 0.517 | 0.04 |
| *Calathus melanocephalus* | 0.005 | 0.979 | <0.01 | 2.769 | 0.184 | 0.08 | 1.754 | 0.362 | 0.02 |
| *Cerapteryx graminis* larva | 1.299 | 0.521 | 0.07 | 3.616 | 0.161 | 0.08 | 2.647 | 0.283 | 0.05 |
| Chironomidae spp. | 1.263 | 0.521 | 0.01 | 0.007 | 0.942 | <0.01 | 0.365 | 0.572 | <0.01 |
| Chironomidae spp. larva | NA | NA | NA | 0.062 | 0.899 | <0.01 | 2.093 | 0.306 | 0.03 |
| Collembola spp. | 5.703 | 0.032 | 0.38 | 5.852 | 0.011 | 0.37 | 1.464 | 0.362 | 0.01 |
| *Cytilus sericeus* | 2.063 | 0.456 | 0.30 | 5.255 | 0.093 | 0.12 | 4.244 | 0.104 | 0.21 |
| *Deroceras* spp. | 2.157 | 0.456 | 0.15 | 1.780 | 0.286 | 0.14 | 0.816 | 0.568 | 0.03 |
| Diptera spp. | 0.001 | 0.979 | <0.01 | 7.019 | 0.004 | 0.42 | 2.563 | 0.283 | 0.05 |
| *Eana osseana* | 1.554 | 0.521 | 0.27 | 2.243 | 0.220 | 0.15 | 0.933 | 0.517 | 0.04 |
| *Gonatium* spp. | 5.501 | 0.221 | 0.23 | 0.005 | 0.942 | <0.01 | 1.529 | 0.362 | 0.02 |
| Hemiptera spp. nymph | 16.44 | <0.001 | 0.65 | 2.359 | 0.245 | 0.04 | 1.951 | 0.345 | 0.03 |
| Hymenoptera spp. | 1.857 | 0.456 | 0.04 | 4.696 | 0.111 | 0.11 | 1.458 | 0.362 | 0.01 |
| *Hypnoidus riparius* | 0.278 | 0.786 | <0.01 | 4.373 | 0.118 | 0.10 | 4.865 | 0.114 | 0.11 |
| *Mitopus morio* | 0.957 | 0.603 | 0.09 | 5.739 | 0.045 | 0.19 | 6.717 | 0.063 | 0.16 |
| Oligochaeta spp. | NA | NA | NA | 1.041 | 0.398 | <0.01 | 2.789 | 0.267 | 0.05 |
| *Pardosa palustris* | 0.356 | 0.845 | <0.01 | 13.31 | 0.001 | 0.46 | 23.76 | <0.001 | 0.42 |
| *Pardosa prativaga* | 1.870 | 0.456 | 0.10 | 0.906 | 0.498 | 0.02 | 2.004 | 0.317 | 0.10 |
| *Patrobus septentrionis* | 2.902 | 0.407 | 0.34 | 8.401 | 0.001 | 0.50 | 10.71 | <0.001 | 0.51 |
| *Pirata piraticus* | 0.533 | 0.646 | <0.01 | 2.397 | 0.184 | 0.15 | 4.695 | 0.028 | 0.32 |
| *Platygastridae* spp. | 9.343 | 0.042 | 0.22 | 1.559 | 0.321 | 0.02 | 0.677 | 0.517 | <0.01 |
| *Pterostichus diligens* | 3.540 | 0.349 | 0.14 | 66.65 | <0.001 | 0.68 | 20.78 | <0.001 | 0.59 |
| *Pterostichus nigrita* | 3.014 | 0.344 | 0.34 | 15.27 | 0.003 | 0.32 | 6.306 | 0.012 | 0.36 |
| *Staphylinidae* spp. | 1.075 | 0.526 | <0.01 | 0.476 | 0.575 | <0.01 | 0.920 | 0.548 | 0.03 |
| *Thysanoptera* spp. nymph | 1.053 | 0.526 | <0.01 | 1.630 | 0.316 | 0.10 | 1.060 | 0.517 | 0.03 |

**Table S5.** *F*, *p*, and *r*^2^ values from the GAM analyses of species richness, Pielou's evenness, Shannon diversity, mean body mass, total abundance, or total biomass of invertebrates sampled in July 2013 as a function of temperature, with species richness, Pielou's evenness, Shannon diversity, or percentage cover of plants included as covariates.

| **Response variable** | **Explanatory variable** | ***F* value** | ***p* value** | ***r*^2^** |
| --- | --- | --- | --- | --- |
| invertebrate richness | plant richness | 0.134 | 0.717 |  |
|  | temperature | 0.190 | 0.666 | <0.01 |
|  |  |  |  |  |
|  | plant evenness | 0.176 | 0.678 |  |
|  | temperature | 0.037 | 0.849 | <0.01 |
|  |  |  |  |  |
|  | plant diversity | 0.216 | 0.645 |  |
|  | temperature | 0.024 | 0.878 | <0.01 |
|  |  |  |  |  |
|  | % cover of plants | 0.475 | 0.496 |  |
|  | temperature | 0.130 | 0.721 | <0.01 |
|  |  |  |  |  |
| invertebrate evenness | plant richness | 1.460 | 0.237 |  |
|  | temperature | 10.380 | 0.001 | 0.38 |
|  |  |  |  |  |
|  | plant evenness | 5.968 | 0.021 |  |
|  | temperature | 25.690 | <0.001 | 0.45 |
|  |  |  |  |  |
|  | plant diversity | 3.322 | 0.079 |  |
|  | temperature | 20.340 | <0.001 | 0.41 |
|  |  |  |  |  |
|  | % cover of plants | 1.057 | 0.313 |  |
|  | temperature | 14.490 | <0.001 | 0.37 |
|  |  |  |  |  |
| invertebrate diversity | plant richness | 2.322 | 0.139 |  |
|  | temperature | 18.620 | <0.001 | 0.36 |
|  |  |  |  |  |
|  | plant evenness | 5.024 | 0.033 |  |
|  | temperature | 21.800 | <0.001 | 0.41 |
|  |  |  |  |  |
|  | plant diversity | 3.268 | 0.081 |  |
|  | temperature | 19.940 | <0.001 | 0.38 |
|  |  |  |  |  |
|  | % cover of plants | 0.587 | 0.450 |  |
|  | temperature | 14.990 | 0.001 | 0.32 |
|  |  |  |  |  |
| mean body mass | plant richness | 0.640 | 0.430 |  |
|  | temperature | 8.234 | 0.008 | 0.18 |
|  |  |  |  |  |
|  | plant evenness | 1.386 | 0.249 |  |
|  | temperature | 9.169 | 0.005 | 0.20 |
|  |  |  |  |  |
|  | plant diversity | 0.834 | 0.369 |  |
|  | temperature | 8.598 | 0.007 | 0.18 |
|  |  |  |  |  |
|  | % cover of plants | 1.217 | 0.279 |  |
|  | temperature | 7.484 | 0.011 | 0.19 |
|  |  |  |  |  |
| total abundance | plant richness | 0.984 | 0.330 |  |
|  | temperature | 6.127 | 0.020 | 0.22 |
|  |  |  |  |  |
|  | plant evenness | 0.138 | 0.713 |  |
|  | temperature | 9.278 | 0.005 | 0.20 |
|  |  |  |  |  |
|  | plant diversity | 0.025 | 0.875 |  |
|  | temperature | 8.090 | 0.008 | 0.20 |
|  |  |  |  |  |
|  | % cover of plants | 1.154 | 0.292 |  |
|  | temperature | 9.290 | 0.005 | 0.23 |
|  |  |  |  |  |
| total biomass | plant richness | 0.496 | 0.184 |  |
|  | temperature | 1.569 | 0.221 | 0.02 |
|  |  |  |  |  |
|  | plant evenness | 0.540 | 0.469 |  |
|  | temperature | 0.880 | 0.356 | <0.01 |
|  |  |  |  |  |
|  | plant diversity | 0.877 | 0.357 |  |
|  | temperature | 1.059 | 0.312 | <0.01 |
|  |  |  |  |  |
|  | % cover of plants | 0.035 | 0.854 |  |
|  | temperature | 0.558 | 0.461 | <0.01 |

**Fig. S1.** Length-weight relationships for terrestrial invertebrate taxa calculated from data collected in August 2012. Log_10_ transformations were applied to both length (mm) and body mass (dry weight in mg) data. Relationships were only defined at the species level when *r*^2^ ≥ 0.7. Relationships at the Family and Order level include the same data from lower levels of taxonomic resolution. The number of data points (*n*) making up each relationship is shown in the bottom right of each panel. See Table S2 for details of linear regression equations and *r*^2^ values.


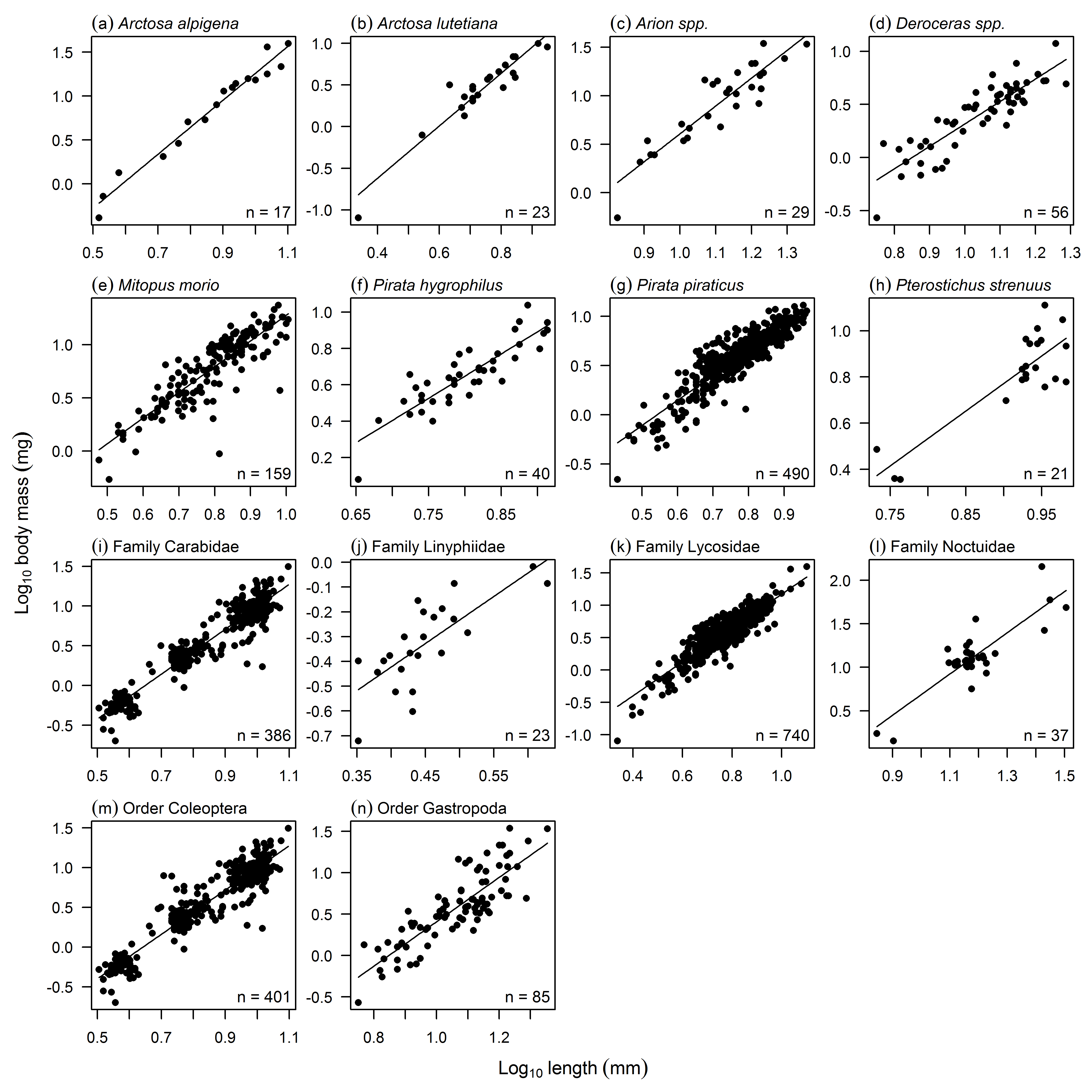


**Fig. S2.** There was no evidence for spatial autocorrelation of model residuals for: (a) plant species richness (Mantel test: *r* = -0.034, *p* = 0.104); (b) invertebrate species richness (Mantel test: *r* = 0.018, *p* = 0.290); (c) Pielou’s evenness for plants (Mantel test: *r* = -0.062, *p* = 0.054); (d) Pielou’s evenness for invertebrates (Mantel test: *r* = -0.006, *p* = 0.284); (e) Shannon diversity for plants (Mantel test: *r* = -0.061, *p* = 0.061); (f) Shannon diversity for invertebrates (Mantel test: *r* = -0.001, *p* = 0.379) in July 2013. Outer lines delineate the 95% confidence intervals, which never cross the zero line in these spline correlograms.


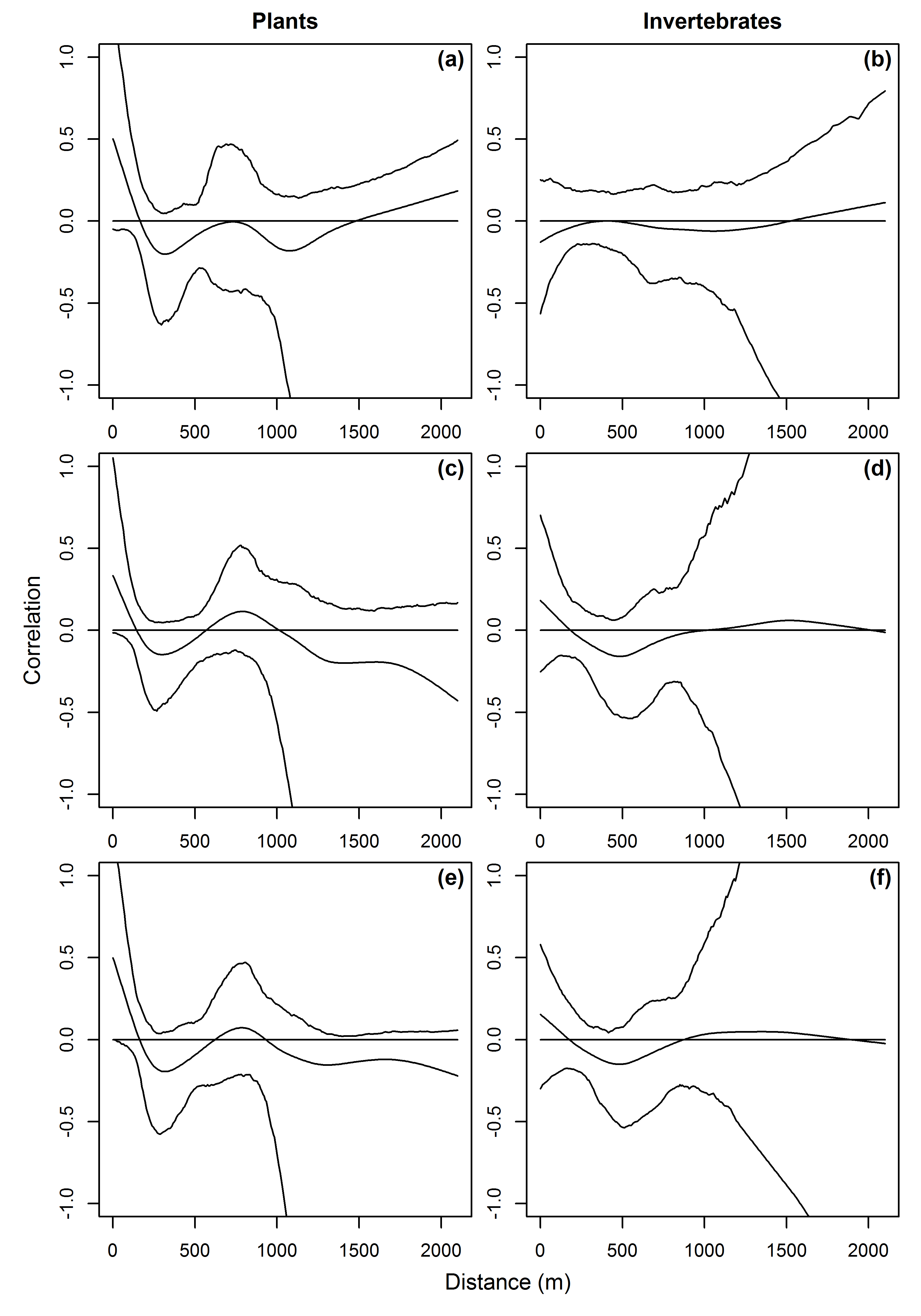


**Fig. S3.** There was little evidence for spatial autocorrelation of model residuals for Sørensen similarity in community composition of: (a) plants (Mantel test: *r* = -0.142, *p* = 0.113); (b) invertebrates (Mantel test: *r* = -0.163, *p* = 0.096) with increasing pairwise temperature difference between sites in July 2013. It should be noted that the spline correlograms indicate that there may be some positive autocorrelation at short distances (0-200 m) for both plants and invertebrates because the outer lines, which delineate the 95% confidence intervals, are both above the zero line. There is also some evidence for negative autocorrelation at distances of around 500-1,000 m for invertebrates, where the 95% confidence intervals are both below the zero line.


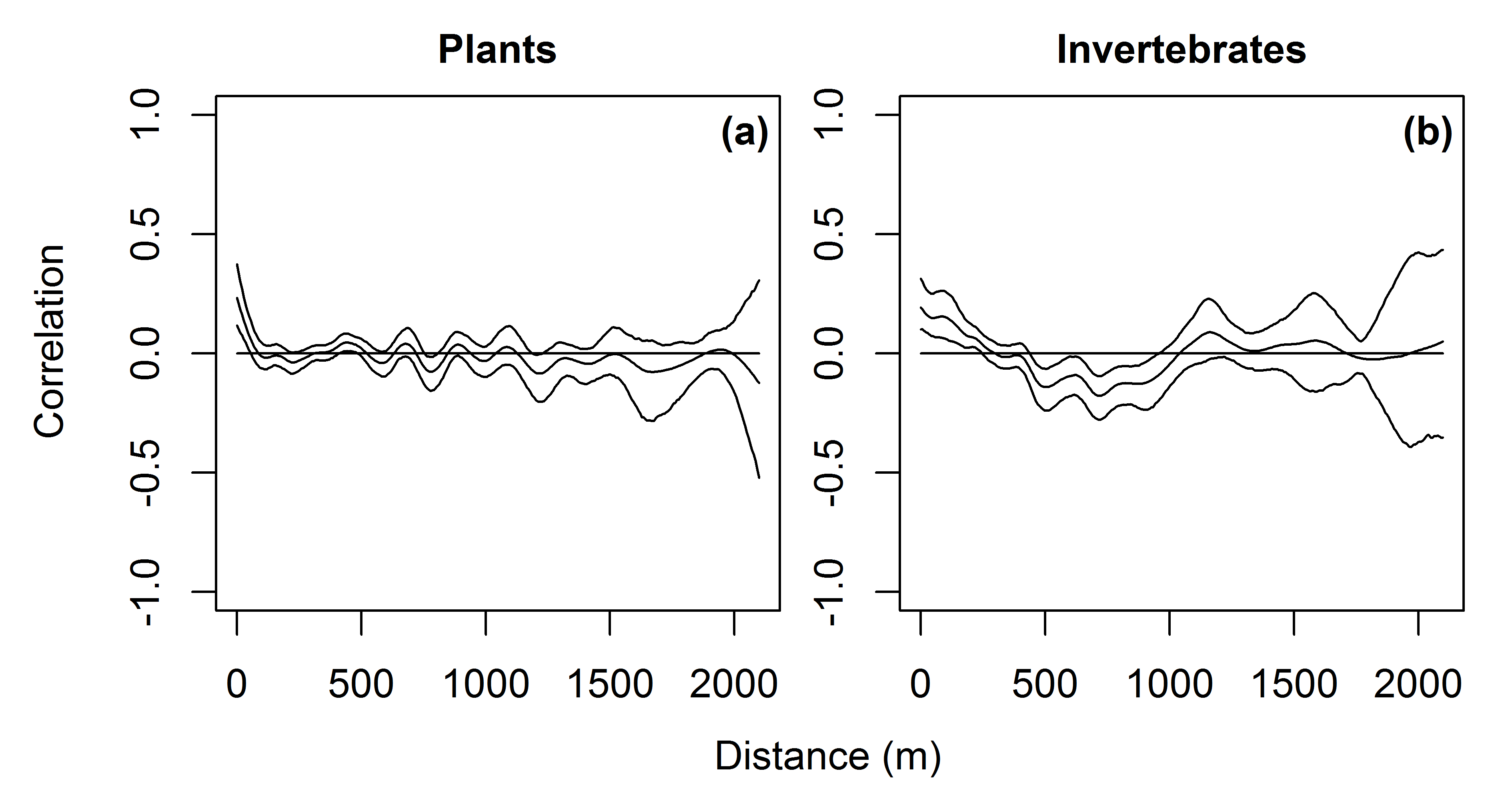


**Fig. S4.** There was no evidence for spatial autocorrelation of model residuals for: (a) mean body mass (Mantel test: *r* = 0.015, *p* = 0.352); (b) total abundance (Mantel test: *r* = -0.036, *p* = 0.121); (c) total biomass (Mantel test= *r* = 0.021, *p* = 0.214) of the invertebrate community in July 2013. Outer lines delineate the 95% confidence intervals, which never cross the zero line in these spline correlograms.


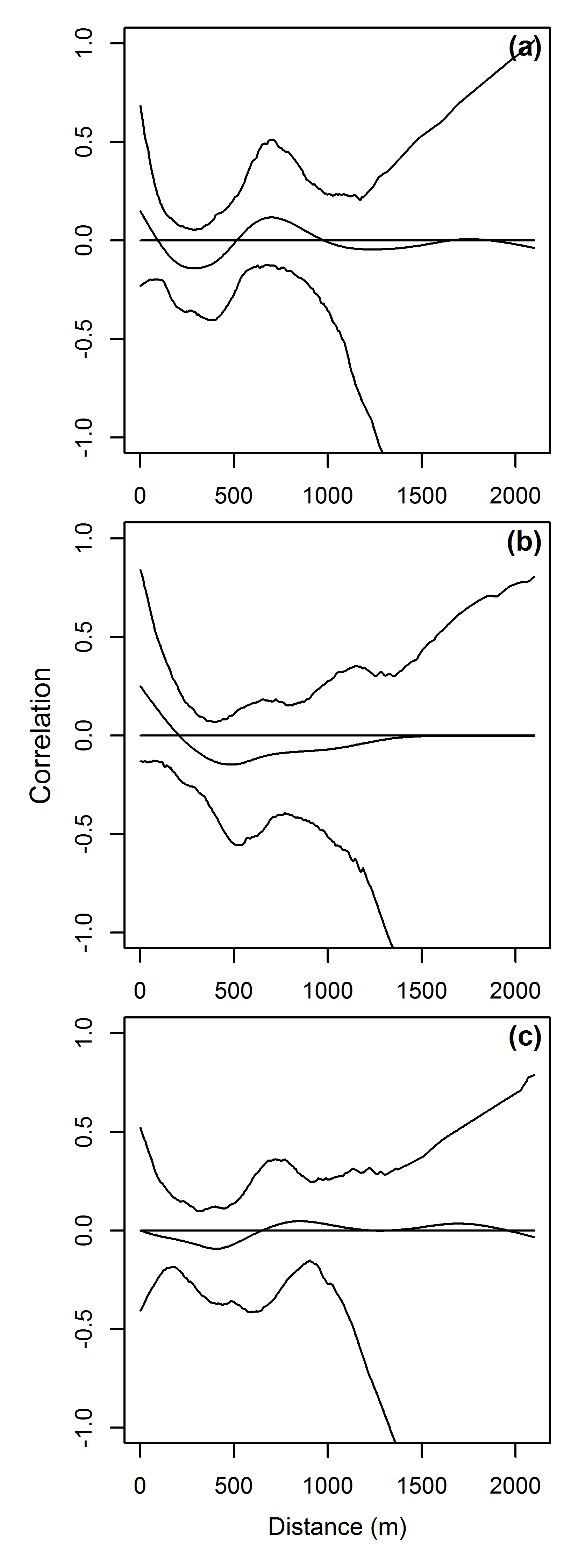


**Fig. S5** Significant effects of temperature on the percentage cover of vegetation species sampled in July 2013. Solid and dashed lines are the predicted fitting and 95% confidence intervals, respectively, from GAM models (see Table S3).

**
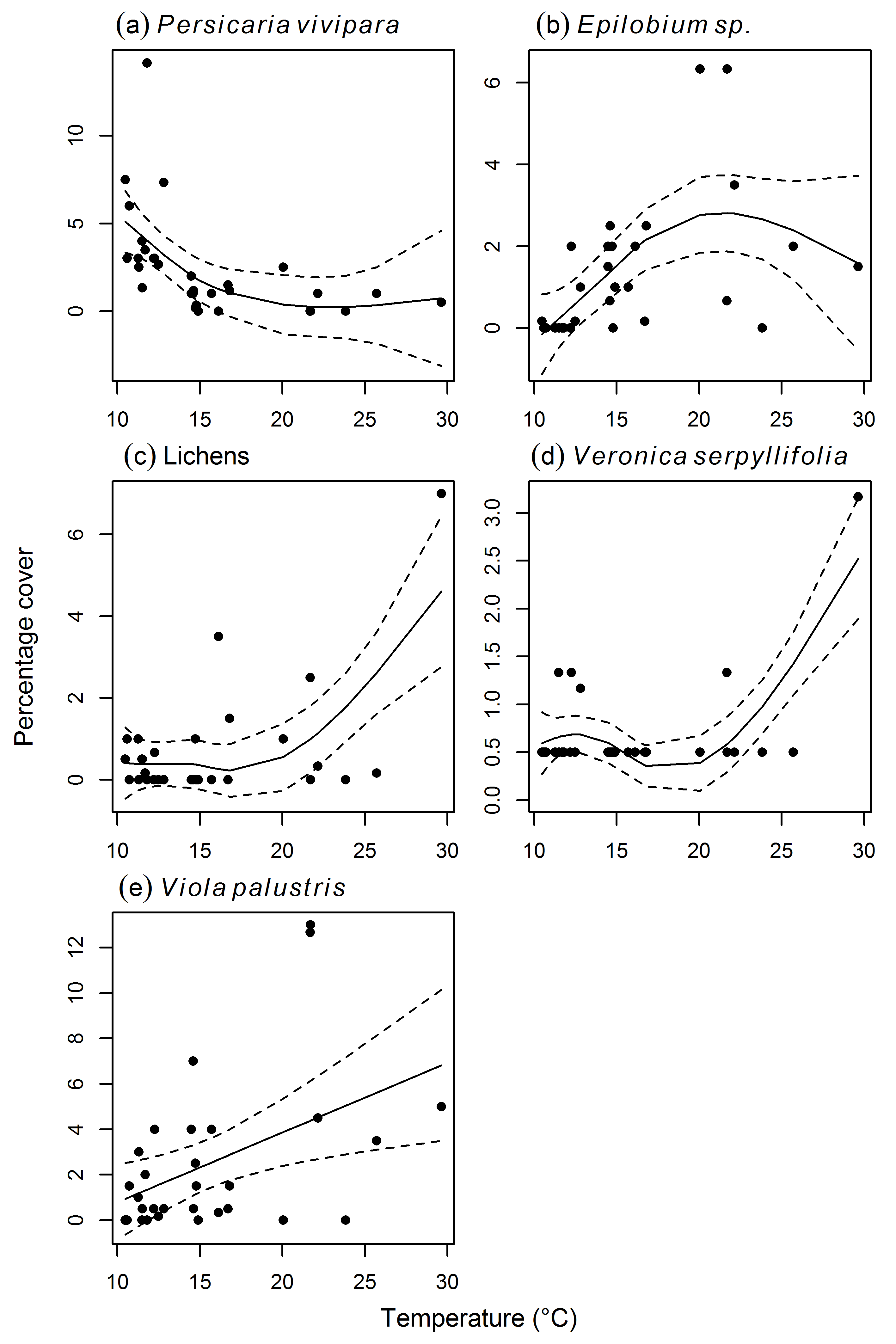
**

**Fig. S6.** Relationships between soil temperature and various metrics of diversity for invertebrates in August 2012 (open circles) and July 2013 (solid circles). All possible combinations of the main and interactive effects of temperature (*T*) and year (*Y*) were explored with ANCOVA and the model with the lowest AIC value was chosen as the best fitting model for each diversity metric: (a) invertebrate species richness (*S* = 10.70 + 4.760*Y*; *r*^2^ = 0.56, *F*_1,62_ = 79.57, *p* < 0.001); (b) Pielou's evenness (*J'* = 0.941 - 0.013*T* – 0.136*Y*; *r*^2^ = 0.68, *F*_2,61_ = 68.51, *p* < 0.001); (c) Shannon diversity (*H'* = 2.220 - 0.035*T*; *r*^2^ = 0.41, *F*_1,62_ = 45.64, *p* < 0.001); (d) Sørensen similarity in invertebrate community composition and pairwise temperature difference between sites in August 2012 (*β* = 0.588 – 0.010*T;* Mantel *r* = 0.355, *p* = 0.001).


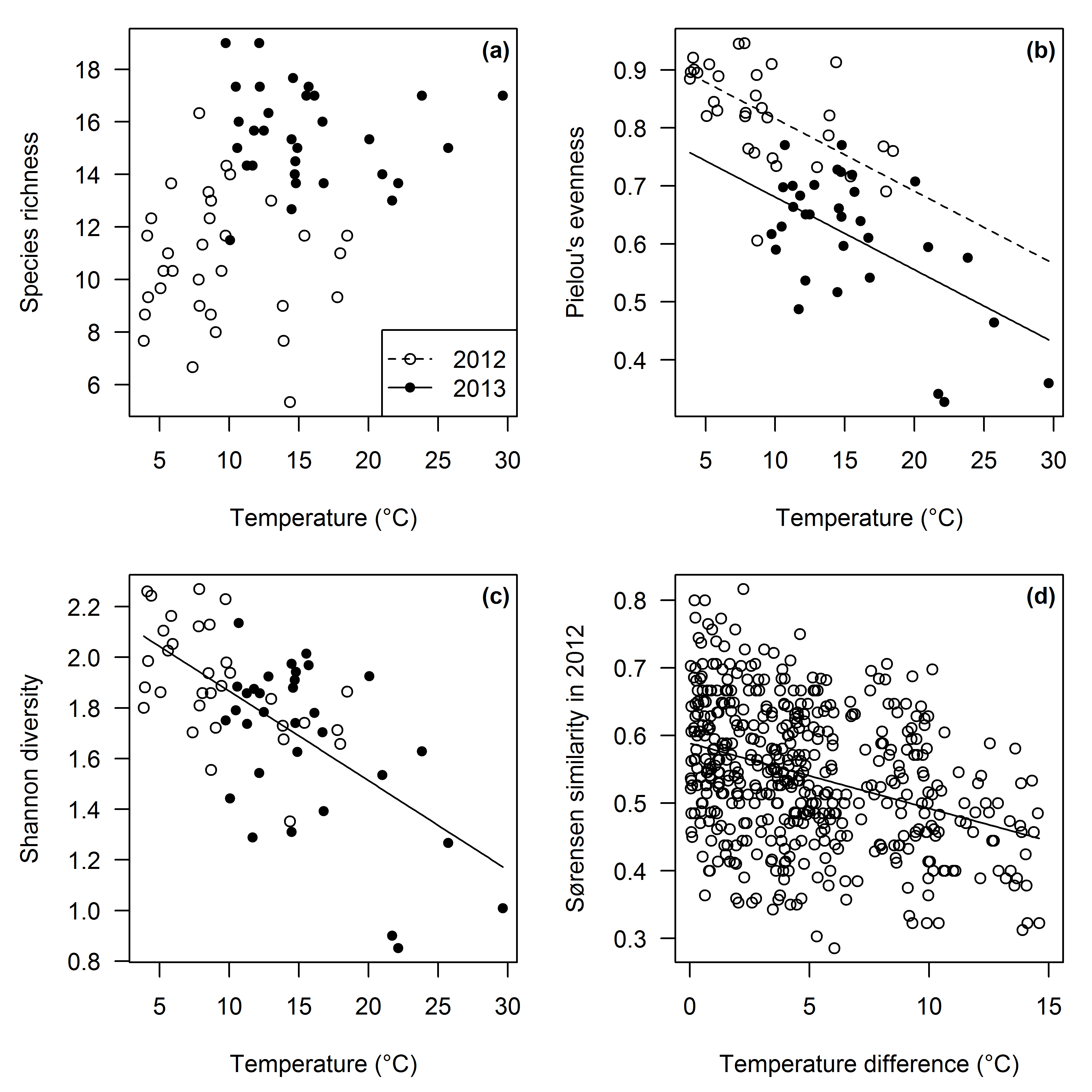


**Fig. S7.** Relationships between soil temperature and invertebrate community metrics in August 2012 (open circles) and July 2013 (solid circles). All possible combinations of the main and interactive effects of temperature (*T*) and year (*Y*) were explored with ANCOVA and the model with the lowest AIC value was chosen as the best fitting model for each metric: (a) mean body mass (*M* = 0.510 - 0.025*T*; *r*^2^ = 0.26, *F*_1,62_ = 23.05, *p* < 0.001); (b) total abundance (*N* = 1.360 + 0.024*T* + 0.412*Y*; *r*^2^ = 0.66, *F*_2,61_ = 61.23, *p* < 0.001); (c) total biomass (*B* = 1.858 + 0.346*Y*; *r*^2^ = 0.40, *F*_1,62_ = 42.13, *p* < 0.001).


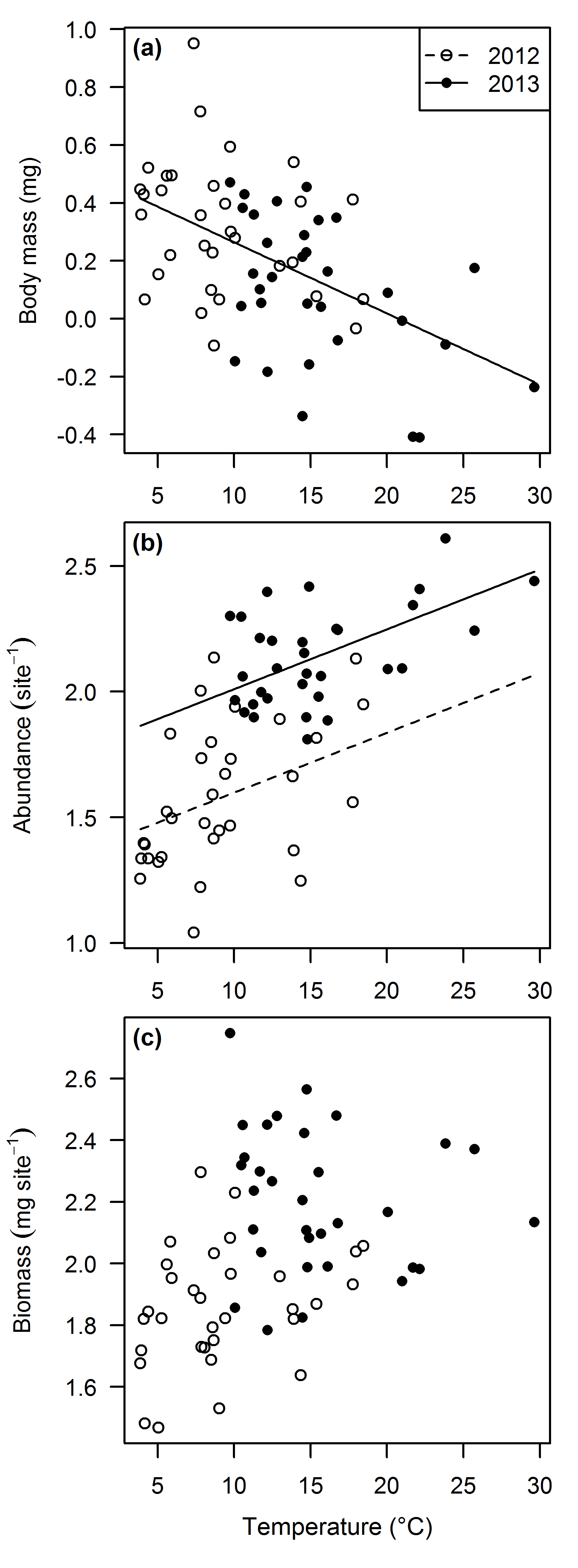


**Fig. S8.** Significant effects of temperature on the mean body mass of invertebrate populations sampled in July 2013. Solid and dashed lines are the predicted fitting and 95% confidence intervals, respectively, from GAM models (see Table S4).


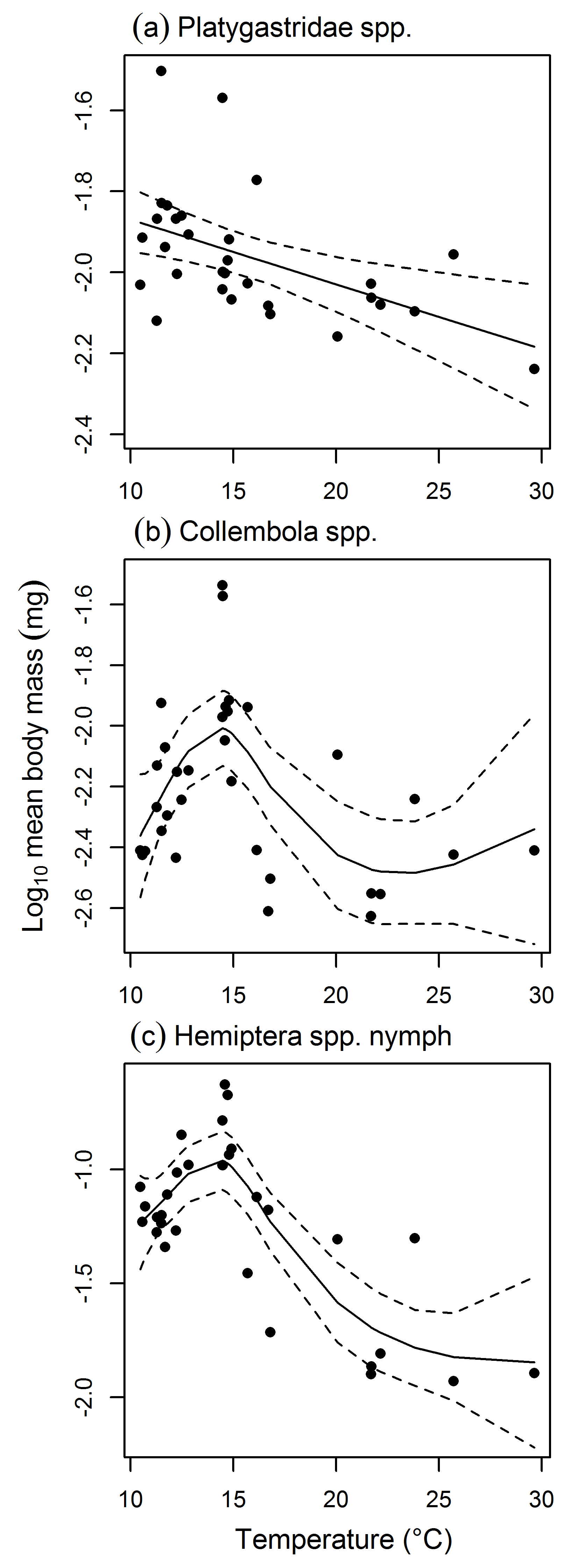


**Fig. S9.** Significant effects of temperature on the total abundance of invertebrate populations sampled in July 2013. Solid and dashed lines are the predicted fitting and 95% confidence intervals, respectively, from GAM models (see Table S4).

**
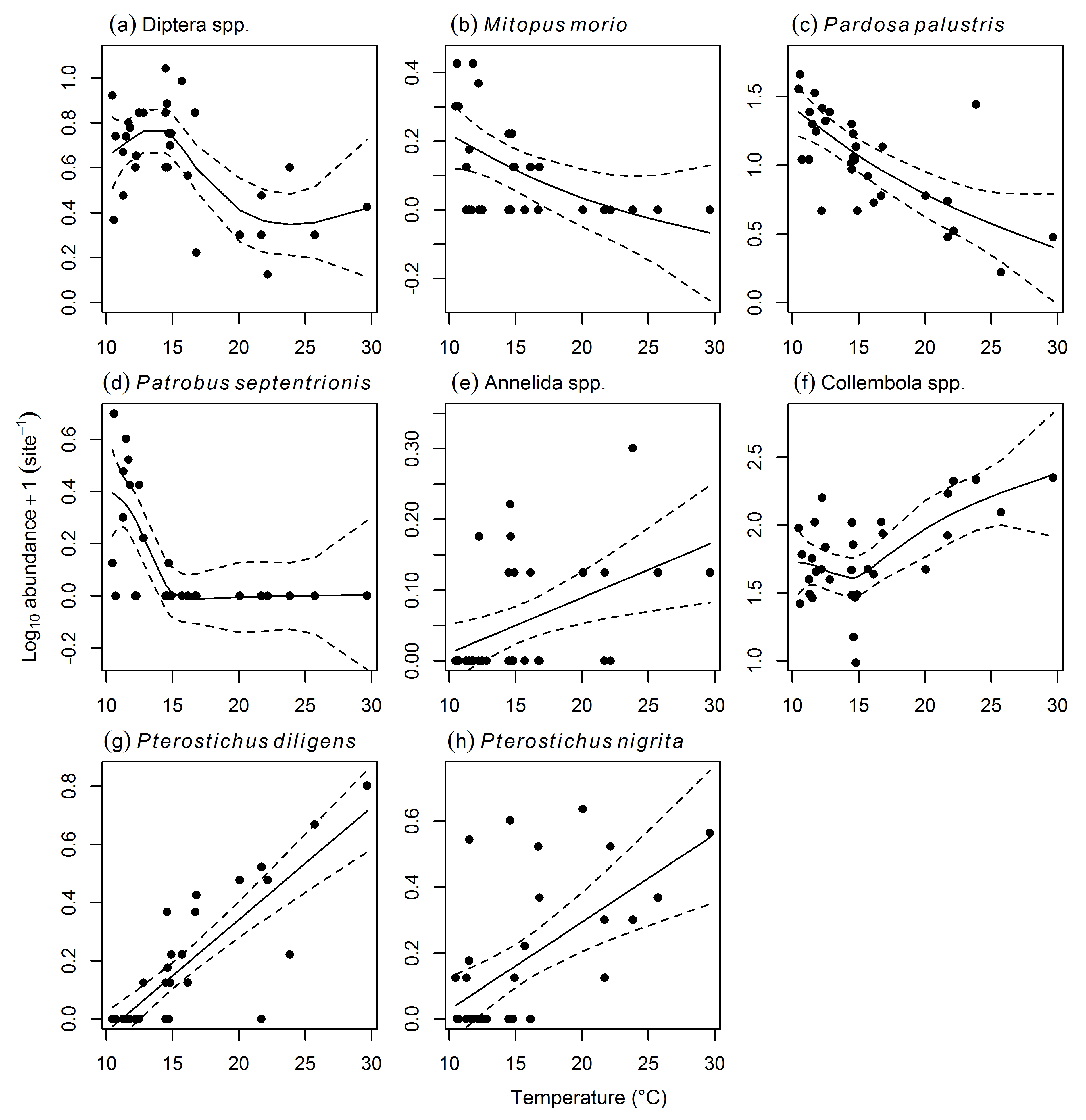
**

**Fig. S10.** Significant effects of temperature on the total biomass of invertebrate populations sampled in July 2013. Solid and dashed lines are the predicted fitting and 95% confidence intervals, respectively, from GAM models (see Table S4).

**
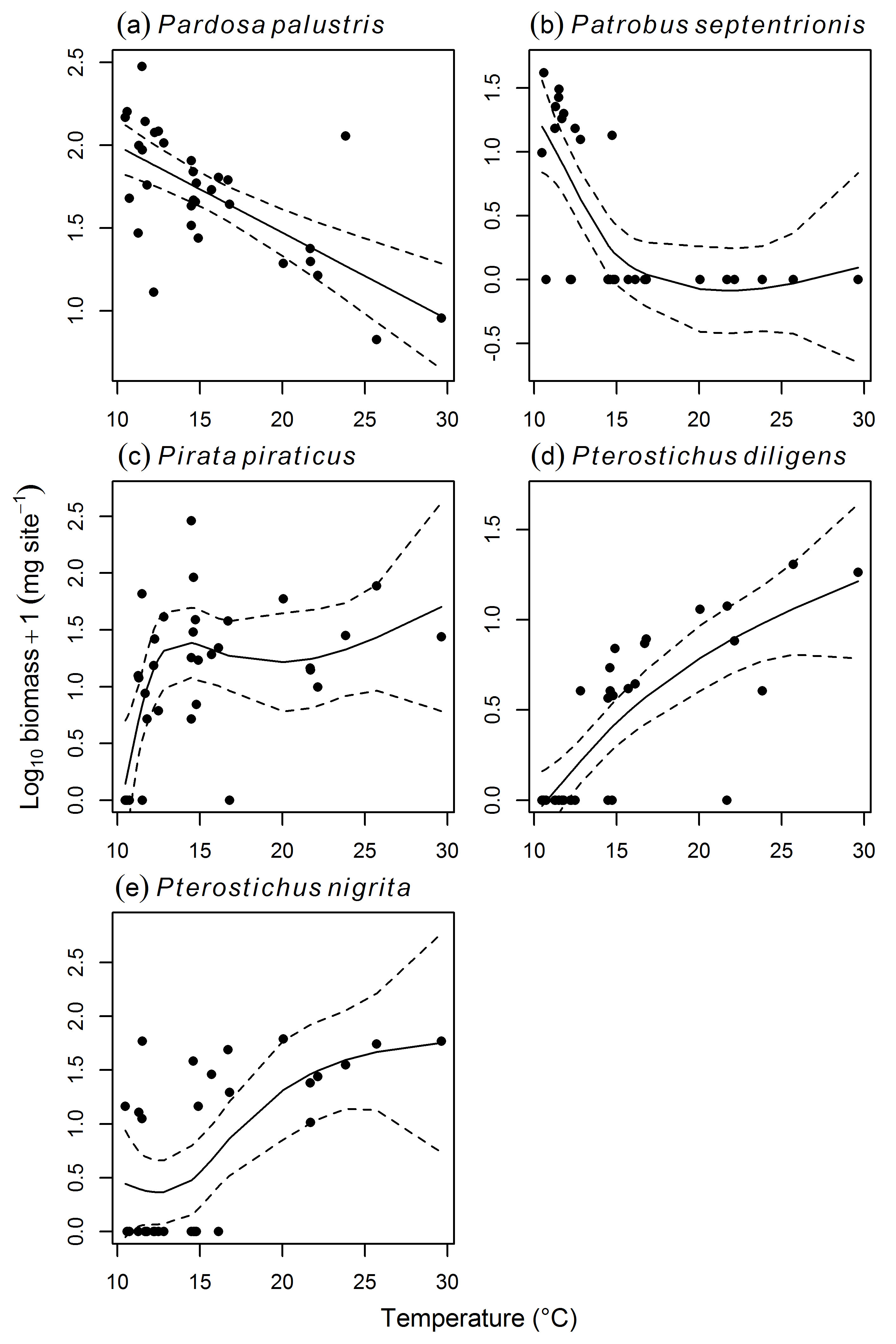
**
